# Artemisinin and its derivatives for metabolic syndrome: From multi-target mechanisms to translational opportunities (Review)

**DOI:** 10.3892/mmr.2026.14026

**Published:** 2026-09-17

**Authors:** Shuo Cao, Mo Yang, Siyu Qu, Jinsong Wu, Jiaheng Li, Ying Zhao

**Affiliations:** 1 College of Traditional Chinese Medicine, Shandong University of Traditional Chinese Medicine, Jinan, Shandong 250355, P.R. China; 2 School of Health, Shandong University of Traditional Chinese Medicine, Jinan, Shandong 250355, P.R. China

**Keywords:** artemisinin, metabolic syndrome, insulin resistance, multi-target mechanisms, inflammation, gut microbiota

## Abstract

Metabolic syndrome (MetS) is a complex of disorders, characterized by insulin resistance accompanied by abdominal obesity, dyslipidemia, hypertension and impaired glucose metabolism. This group of conditions notably increases the risk of type 2 diabetes mellitus and cardiovascular or cerebrovascular diseases and has become a major global public health challenge. Current treatments for MetS often fail to meet clinical needs owing to the limitations associated with single-target therapies and notable side effects. Artemisinin-based drugs are primarily used as antimalarial drugs but have garnered widespread attention for their broad biological activities. Preclinical evidence suggests that artemisinin and its derivatives improve insulin sensitivity, regulate lipid metabolism, alleviate chronic inflammation and reshape the gut microbiota by modulating multiple metabolism-related signaling pathways, including AMP-activated protein kinase, PI3K/Akt, NF-κB and Toll-like receptor 4, indicating a potential therapeutic avenue for MetS. However, most of these findings are derived from cell-based and animal studies, while direct clinical evidence in patients with MetS remains limited. In the present review, the therapeutic potential and core molecular mechanisms of artemisinin and its derivatives for MetS are summarized and the challenges and opportunities for their future clinical translation are discussed.

## 1. Introduction

Metabolic syndrome (MetS) is defined by the World Health Organization (WHO) as a cluster of disorders characterized by insulin resistance accompanied by multiple abnormalities such as abdominal obesity, dyslipidemia, hypertension and hyperglycemia (1). This complex condition notably increases the risk of type 2 diabetes mellitus (T2DM), cardiovascular disease, non-alcoholic fatty liver disease (NAFLD) and certain cancer types (2–4). MetS represents a major global public-health challenge, affecting ~20% of the global population, with prevalence rates reaching up to 46% in high-burden geographical regions, such as the Middle-East and Latin-America, and a rising prevalence observed among younger age groups, namely adolescents and young adults aged 10–24 years (5,6). Insulin resistance is the central pathological mechanism of MetS, directly leading to impaired glucose metabolism and compensatory hyperinsulinemia (7). Dyslipidemia promotes the release of large amounts of pro-inflammatory factors from macrophages in adipose tissue, which inhibit insulin signaling and promote atherosclerosis (8). Oxidative stress leads to the accumulation of reactive oxygen species (ROS), damaging cellular structures and mutually amplifying inflammation (9,10). Dysbiosis of the gut microbiota disrupts the intestinal barrier, allowing endotoxins to enter the bloodstream, thereby activating systemic inflammation and disrupting glucose and lipid metabolism (11,12). These mechanisms are interlinked, creating a vicious cycle that ultimately leads to the coexistence of various abnormalities, including disorders involving glucose and lipid metabolism, obesity and hypertension.

Pharmacotherapies currently used for MetS management are unable to target its full spectrum of pathological features and have been associated with notable concerns regarding adverse effects. For instance, although metformin can improve insulin resistance, the effects of metformin on lipid metabolism disorders and abdominal obesity are limited, and some patients discontinue the medication after developing gastrointestinal side effects (13). Sodium-glucose cotransporter 2 (SGLT2) inhibitors promote weight loss and protect renal function but increase the risk of urinary tract infections (14). Therefore, a metabolic modulator that acts on multiple disease-related pathways with a favorable safety profile is urgently needed. Artemisinin is derived from the traditional Chinese medicinal plant *Artemisia annua*. Since Tu Youyou's group discovered the antimalarial activity of artemisinin and was awarded the Nobel Prize in 2015, artemisinin-based drugs have become the global first-line treatment for malaria (15). Previous research has revealed that the biological activities of artemisinin and its derivatives may extend far beyond antimalarial therapy, and have been reported to demonstrate antitumor, cardioprotective, antifibrotic and antioxidant effects (16–19).

In the context of MetS, artemisinin and its derivatives have been reported to improve metabolic disorders through effects on obesity, insulin resistance, inflammation, the gut microbiota and lipid metabolism (20–25). Furthermore, these compounds are generally well-tolerated at conventional therapeutic doses in short-term use, while long-term safety data for chronic metabolic indications remain limited. However, although artemisinin-based drugs have been shown to affect multiple components of MetS, suggesting that these drugs may address multiple pathological features of MetS, the specific regulatory mechanisms of artemisinin-based drugs have not been fully elucidated, and clinical translation requires long-term evidence of safety and efficacy (16). In the present review, the scientific basis for the potential transition of artemisinin from a leading antimalarial agent to a metabolic modulator is summarized, providing an in-depth assessment of artemisinin signaling networks and molecular mechanisms to offer theoretical support and future research directions for developing prevention and treatment strategies for MetS.

## 2. Structural basis and pharmacological effects of artemisinin and its derivatives

Artemisinin is a sesquiterpene lactone compound that occurs naturally in the glandular trichomes of *A. annua* (family: Asteraceae) and is an active ingredient in traditional Chinese medicine (26,27). Artemisinin has the molecular formula C_15_H_22_O_5_ and a molecular weight of 282.33 g/mol. The core structure of artemisinin consists of a 15-carbon sesquiterpene skeleton containing a lactone ring and a 1,2,4-trioxane endoperoxide bridge, which serves as the key pharmacophore responsible for the antimalarial and other biological activities of artemisinin (28).

Structurally, the regulatory effects of artemisinin and its derivatives on signaling pathways are rooted in two core features: The endoperoxide bridge and the C-10 position, which together define the bioactivity and target selectivity of artemisinin and its derivatives. The endoperoxide bridge serves as the principal pharmacophoric trigger. The reductive cleavage of the endoperoxide bridge generates carbon-centered free radicals and ROS, which elevate the AMP/ATP ratio and activate AMP-activated protein kinase (AMPK), leading to mTOR suppression, which in turn promotes autophagy, while also inhibiting NF-κB-driven inflammation. In parallel, endoperoxide-derived reactive species modulate upstream signaling components such as inhibitor of NF-κB kinase, while direct myeloid differentiation protein 2 (MD2) binding may disrupt toll-like receptor 4 (TLR4)/MD2 complex formation, with both mechanisms converging on NF-κB repression (29,30). The C-10 position, in turn, acts as a key structural determinant of potency and selectivity. Structure-activity relationship studies have demonstrated that C-10 substituents modulate TLR4/NF-κB inhibitory activity in a chain-length-dependent bell-shaped manner, with optimal efficacy at the 3,4 carbon atoms. Variations in C-10 substituents (hydroxyl, methyl ether or hemisuccinate ester) alter the physicochemical properties and target-binding affinity of the derivatives, thereby fine-tuning their regulatory capacity on inflammatory signaling pathways (31,32).

Given these unique structural features, artemisinin exhibits broad biological activity. In addition to the role of artemisinin as a first-line antimalarial agent, previous studies have confirmed that artemisinin can target core components of MetS, such as obesity, T2DM, insulin resistance and immune dysfunction, demonstrating the potential of artemisinin for treating multiple aspects of MetS (33,34). In terms of regulating glucose and lipid metabolism, artemisinin modulates glucose transport and utilization and markedly improves insulin sensitivity (21). In a model of non-alcoholic steatohepatitis (NASH), artemisinin restores hepatic lipid homeostasis by regulating transcription factors, such as peroxisome proliferator-activated receptor α (PPARα), alleviates hepatic steatosis and re-establishes lipid homeostasis (35,36). In terms of anti-inflammatory and immunomodulatory effects, artemisinin can suppress inflammatory pathways, reduce the expression levels of pro-inflammatory factors and simultaneously regulate macrophage polarization and regulatory T cell (Treg) differentiation, thereby alleviating inflammatory responses in models of atherosclerosis and ulcerative colitis (37–39). Furthermore, artemisinin-based drugs exert beneficial regulatory effects on the gut microbiota. In a dextran sulfate sodium-induced murine model of ulcerative colitis, dihydroartemisinin (DHA) restores gut microbiota diversity, promotes the enrichment of butyrate-producing bacteria and enhances intestinal barrier function while alleviating inflammation via the gut microbiota-butyrate-Treg/T helper 17 cell (Th17) axis (40).

Owing to drug development bottlenecks, such as the poor aqueous solubility and low oral bioavailability of natural artemisinin, researchers have developed a series of derivatives via structural modification at the C-10 key site. The primary derivatives include: i) DHA, obtained by reducing the C-10 ketone group of artemisinin to a hydroxyl group; ii) Artemether, the C-10 methyl ether derivative of DHA with enhanced lipophilicity; and iii) Artesunate, the C-10 hemisuccinate ester of DHA, featuring high water solubility (41). According to the WHO guidelines for the treatment of malaria, parenteral artesunate is the preferred treatment for severe malaria (42), whereas artemether is usually considered an alternative when artesunate is not available (43). Beyond the licensed use of artemether against uncomplicated malaria in the Food and Drug Administration (FDA)-approved artemether/lumefantrine fixed-dose combination, artemether also exerts independent metabolic regulatory effects in a preclinical model, including ameliorating insulin resistance and normalizing glucose and lipid homeostasis (44,45). While mild gastrointestinal suppression was occasionally observed at high doses, no severe adverse effects were reported in these preclinical studies. It should be emphasized that the long-term effects of artemether were not investigated in these studies, and thus remain unknown (24). DHA inhibits abnormal aerobic glycolysis in tumor cells via a Myc proto-oncogene-dependent pathway, demonstrating the capacity of artemisinin derivatives to modulate pathological glucose metabolism (46).

In summary, artemisinin and its derivatives affect the core pathological pathways of MetS through the aforementioned endoperoxide bridge-driven AMPK-mTOR-mediated autophagy induction and NF-κB/TLR4/MD2 inflammatory repression, as well as C-10 substituent-modulated inflammatory-signaling mechanisms, offering multiple metabolic benefits. These multifaceted biological activities provide a strong rationale for further investigating the therapeutic potential of artemisinin-based drugs in metabolic diseases.

## 3. Mechanisms of artemisinin and its derivatives in regulating MetS

### Regulation of glucose metabolism and insulin resistance

**Figure 1.**
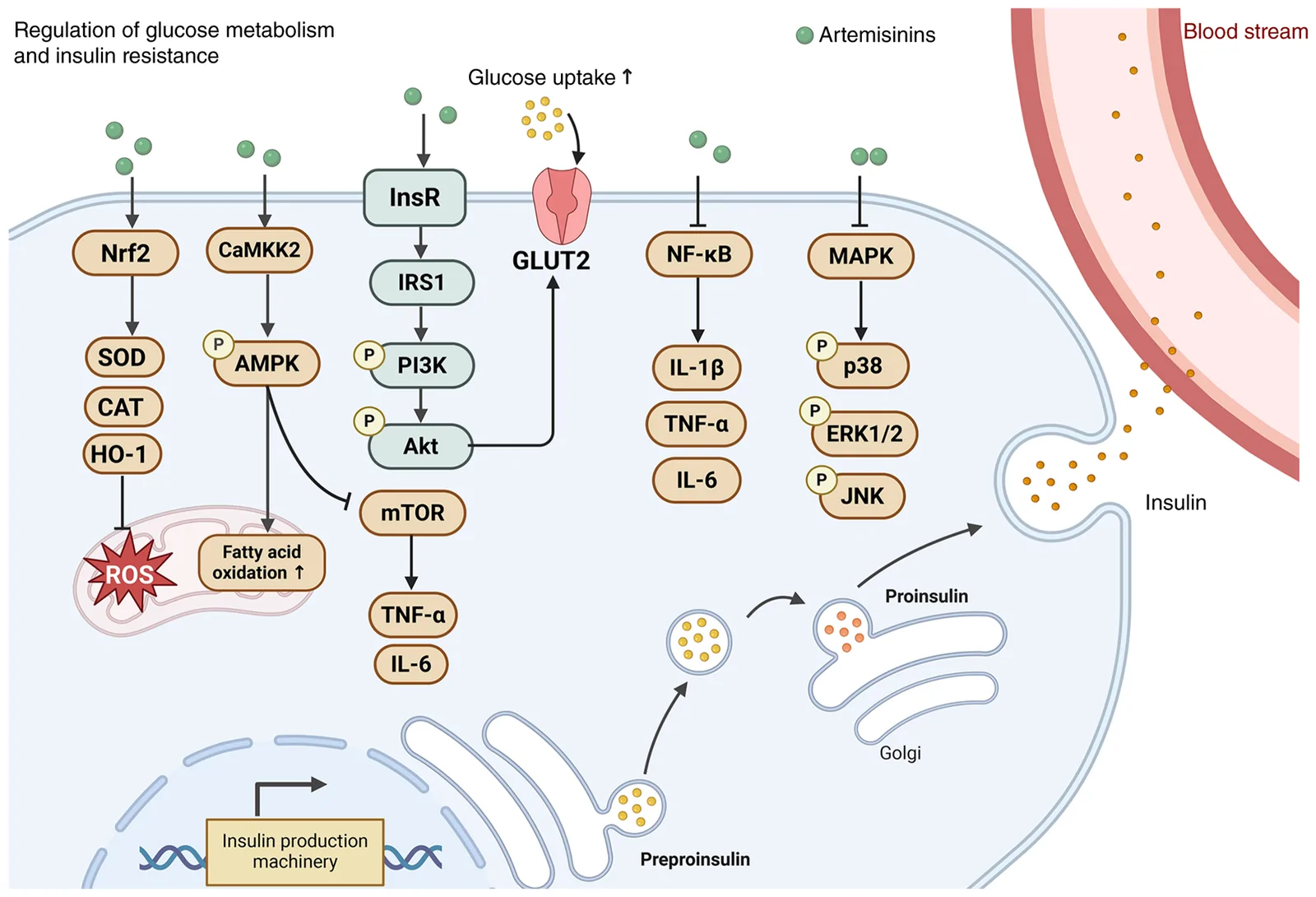
Artemisinin and its derivatives enhance glucose uptake and fatty acid oxidation via the CaMKK2/AMPK and PI3K/Akt pathways, while suppressing MAPK-driven inflammation to improve insulin sensitivity. Artemisinin and its derivatives activate CaMKK2/AMPK to promote glucose uptake and fatty acid oxidation and stimulate PI3K/Akt to facilitate glucose transport and glycogen synthesis. Artemisinin and its derivatives also inhibit MAPK signaling by reducing p38, ERK1/2 and JNK phosphorylation, attenuating insulin resistance. Additionally, artemisinin and its derivatives activate Nrf2 to upregulate antioxidant enzymes and support insulin production. This figure provides a general overview of the shared mechanisms of artemisinin and its derivatives across multiple tissues. Created with BioRender.com. AMPK, AMP-activated protein kinase; CaMKK2, calcium/calmodulin-dependent protein kinase kinase 2; CAT, catalase; GLUT2, glucose transporter type 2; HO-1, heme oxygenase-1; InsR, insulin receptor; IRS1, insulin receptor substrate 1; Nrf2, nuclear factor erythroid 2-related factor 2; p38, p38 mitogen-activated protein kinase; ROS, reactive oxygen species; SOD, superoxide dismutase.

In MetS, reduced insulin sensitivity in organs, such as the liver, notably decreases tissue glucose uptake and utilization alongside abnormal increases in hepatic glucose output and impaired peripheral glucose utilization, ultimately resulting in abnormal blood glucose levels and impaired glucose tolerance (47). Artemisinin and its derivatives directly improve glycemic parameters and enhance insulin signaling by activating cellular energy sensing. Simultaneously, artemisinin and its derivatives inhibit inflammatory and oxidative stress signaling cascades, thereby blocking chronic inflammation-mediated damage to insulin signaling. These effects contribute to comprehensively improving insulin resistance and restoring glucose homeostasis (Fig. 1).

### Liver

In db/db mice, artemisinin activates the hepatic PI3K/Akt pathway, upregulating phosphorylated (p-)PI3K, p-Akt, insulin receptor substrate 1 (IRS1) and glucose transporter type 2 (GLUT2) to enhance glucose uptake and suppress gluconeogenesis. Additionally, artemisinin suppresses MAPK signaling by decreasing phosphorylation of p38 mitogen-activated protein kinase (p38), ERK1/2 and JNK, thus alleviating inflammation- and oxidative stress-triggered insulin resistance (48). Artemisinin also inhibits aberrant glycolysis through regulation of the PI3K/Akt/mTOR axis, thereby preserving metabolic homeostasis. In the liver, artemisinin improves insulin sensitivity and reduces ectopic lipid deposition via the AMPK/nuclear factor erythroid 2-related factor 2 (Nrf2) pathway. At the cellular level, in hepatocytes, artemisinin potentiates IRS1 activity and PI3K/Akt signaling, promotes glucose transport and glycogen synthesis and suppresses the NF-κB inflammatory pathway, leading to reduced expression levels of pro-inflammatory cytokines such as TNF-α, IL-1β and IL-6, which otherwise disrupt insulin signaling (49,50).

### Pancreas

Multiple animal studies have demonstrated that artemisinin-based drugs can considerably reduce fasting and postprandial blood glucose levels (48,51). For instance, artemether and dihydroartemether notably reduced 2 h blood glucose levels in intraperitoneal glucose tolerance and intraperitoneal insulin tolerance tests in a T2DM mouse model. Artemisinin-based drugs also improved serum insulin levels, indicating that these drugs can systemically restore glucose tolerance and insulin sensitivity rather than merely lowering blood glucose levels (21). Artemisinin is hypothesized to induce pancreatic β-cell regeneration to alleviate hyperglycemia, offering a potential strategy to restore pancreatic endocrine function. However, controversy remains regarding this regenerative effect, as multiple studies have failed to replicate α-to-β cell reprogramming in mammalian islet models, highlighting the need for further mechanistic validation (52).

### Adipose tissue

Consistent with the effect of artemether on glycemic control, in a high-fat diet (HFD)-induced obese mouse model, artemether effectively reduced body weight and enhanced systemic insulin sensitivity and glucose tolerance, suggesting that the hypoglycemic effect of artemether reflects a direct improvement in insulin signaling rather than a mere glucose-lowering action (20).

### Systemic metabolic tissues

At the systemic level, artemisinin indirectly improves the microenvironment associated with insulin resistance by inhibiting the mTOR pathway, downregulating pro-inflammatory factors (such as TNF-α and IL-6) and alleviating oxidative stress (53,54). Artemisinin also activates the AMPK pathway by upregulating calcium/calmodulin-dependent protein kinase 2, thereby promoting AMPK phosphorylation and consequently enhancing glucose uptake and fatty acid oxidation. By interacting with the Nrf2 antioxidant axis, artemisinin activates downstream antioxidant enzymes, such as superoxide dismutase (SOD), catalase and heme oxygenase-1, thereby mitigating the damage from oxidative stress induced by high glucose or drug treatments, such as amiodarone-triggered insults in human epithelial cell models. Nrf2-knockdown experiments further confirmed the necessity of artemisinin in maintaining insulin signaling (55,56).

### Non-MetS disease models

Of note, in uveal melanoma cells, artemisinin exerts an opposing effect on the PI3K/Akt/mTOR axis: Artemisinin inhibits the phosphorylation of PI3K, Akt and mTOR, and Akt/mTOR activators can counteract the inhibitory effect of artemisinin on cell migration, demonstrating that artemisinin suppresses cancer cell migration through inhibition of this pathway, highlighting that artemisinin serves as a context-dependent modulator, exerting differential regulatory effects across distinct tissues and pathological states (57).

Given these findings, high-quality clinical studies are needed to clarify the hypoglycemic and insulin-sensitizing efficacy and safety of artemisinin-based drugs in patients with MetS. Artemisinin-based combination regimens should also be explored with metformin and statins to enhance therapeutic efficacy, facilitating the translation of artemisinin derivatives into potential therapeutic agents for MetS.

### Regulation of lipid metabolism and hepatic steatosis

**Figure 2.**
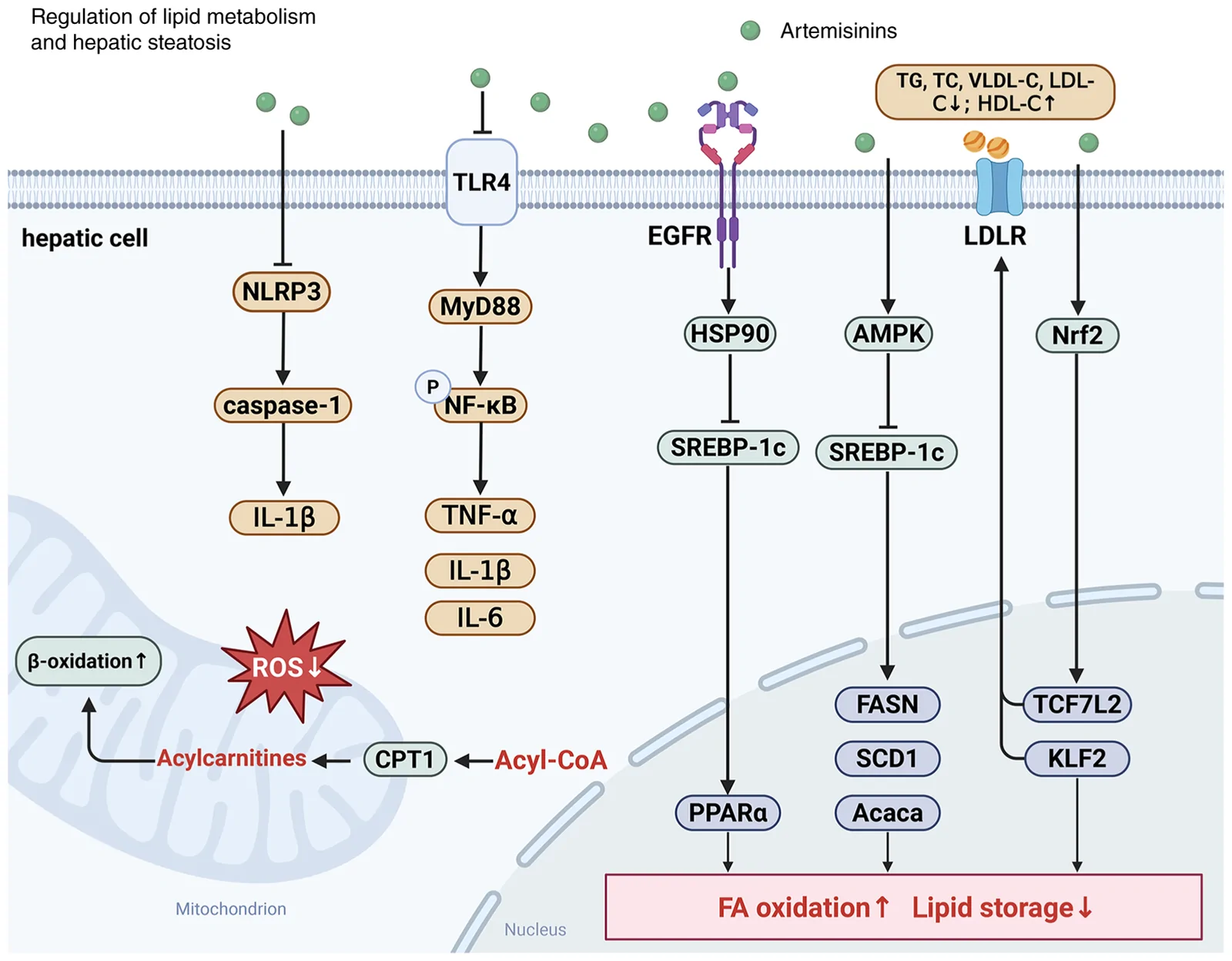
Artemisinin and its derivatives ameliorate hepatic steatosis by activating the AMPK/SREBP-1c and PPARα pathways to suppress *de novo* lipogenesis while concurrently enhancing fatty acid oxidation and reducing lipid storage. Artemisinin and its derivatives also inhibit TLR4/MyD88/NF-κB and NLRP3 inflammasome signaling to attenuate hepatic inflammation. Collectively, these actions lower serum lipids, restore lipid homeostasis and alleviate hepatic steatosis. Created with BioRender.com. Acaca, acetyl-CoA carboxylase α; AMPK, AMP-activated protein kinase; CPT1, carnitine palmitoyltransferase 1; FASN, fatty acid synthase; HDL-C, high-density lipoprotein cholesterol; HSP90, heat shock protein 90; KLF2, Krüppel-like factor 2; LDL-C, low-density lipoprotein cholesterol; LDLR, low-density lipoprotein receptor; MyD88, myeloid differentiation primary response 88; NLRP, nucleotide-binding oligomerization domain, leucine-rich repeat and pyrin domain-containing protein; Nrf2, nuclear factor erythroid 2-related factor 2; PPARα, peroxisome proliferator-activated receptor α; ROS, reactive oxygen species, SCD1, stearoyl-CoA desaturase 1; SREBP-1c, sterol regulatory element-binding protein 1c; TC, total cholesterol; TCF7L2, transcription factor 7-like 2; TG, triglyceride; TLR4, Toll-like receptor 4; VLDL-C, very low-density lipoprotein cholesterol.

Dyslipidemia and hepatic steatosis are interrelated and together constitute a vicious cycle of lipid overload-insulin resistance-chronic inflammation-liver damage (58,59). Artemisinin and its derivatives inhibit hepatic lipid synthesis and promote fatty acid oxidation through effects on several pathways, correcting imbalances involving lipid absorption, synthesis and transport. By modulating the gut-liver axis, artemisinin and its derivatives improve lipid profiles while exerting anti-inflammatory and antioxidant effects and restoring insulin signaling pathways, comprehensively ameliorating lipid metabolism disorders and reversing hepatic steatosis (Fig. 2).

### Liver

In the liver, artemether can reduce the endogenous production of fatty acids and cholesterol by activating the AMPK signaling pathway to inhibit sterol regulatory element-binding protein-1c (SREBP-1c) and its downstream key lipogenic enzymes (acetyl-CoA carboxylase α, fatty acid synthase and stearoyl-CoA desaturase 1), suggesting that artemether can reduce hepatic lipid accumulation by inhibiting excessive lipid synthesis at the source (60). Meanwhile, artesunate notably reduces serum triglyceride (TG) levels and increases high-density lipoprotein cholesterol (HDL-C) in apolipoprotein E-knockout (ApoE^−^/^−^) mice induced by a HFD (61). Regarding the reparative effects on hepatic steatosis, artemether targets the EGFR/heat shock protein 90 (HSP90) complex and upregulates the PPARα/carnitine palmitoyltransferase 1 (CPT1) axis to promote fatty acid β-oxidation, thereby regulating hepatic lipid metabolism and alleviating NASH (60,62,63). Furthermore, artemether can modulate the gut-liver axis, reshape the gut microbiota structure and reduce endotoxemia. By inhibiting the TLR4/NF-κB pathway, artemether blocks activation signals in Kupffer cells, reducing their secretion of pro-inflammatory factors such as TNF-α, IL-1β and IL-6. This action effectively interrupts the amplification of the hepatic inflammatory cascade, ultimately alleviating inflammatory liver damage in NASH (64). Beyond the aforementioned lipid-modulating effects, artemisinin derivatives regulate peroxisome proliferator-activated receptor γ (PPARγ)-mediated lipid metabolic pathways to rebuild the homeostasis of hepatic lipid metabolism and inflammation. Such combined effects ameliorate hepatic lipid deposition and metabolic inflammation synchronously (65). Distinct from these artemether-mediated mechanisms, DHA activates the Nrf2 pathway, inhibits ferroptosis, improves mitochondrial function and indirectly promotes lipid droplet clearance, alleviating hepatic steatosis, inflammation and fibrosis (66).

### Vascular tissue

In atherosclerosis models, artesunate reduces plasma TG, total cholesterol (TC), low-density lipoprotein (LDL) cholesterol (LDL-C), very-LDL-C and arterial lipid deposition through activation of the Krüppel-like factor 2 (KLF2)/Nrf2/transcription factor 7-like 2 (TCF7L2) axis, which enhances LDL receptor-related pathway activity (22). Artemisinin inhibits NOD-like receptor family pyrin domain-containing 3 (NLRP3) inflammasome activation in vascular tissue, reducing caspase-1 and IL-1β expression and decreasing macrophage infiltration and CD68 deposition in arterial plaques. Artemisinin also suppresses NF-κB and other pro-inflammatory signals by regulating the MAPK, PI3K/Akt/mTOR and Nrf2/glutathione (GSH) peroxidase 4 pathways, thereby alleviating vascular chronic inflammation and directly attenuating atherosclerosis (54,61).

### Systemic metabolic tissues

Notably, the lipid-regulating effects of artesunate on TC and LDL appear to be model-dependent. In ApoE^−^/^−^ mice fed a HFD, artesunate markedly reduced atherosclerotic plaque formation and serum TG levels, but did not notably alter TC or LDL levels (61). By contrast, in a study using Wistar rats with normal ApoE function, in which acute atherosclerosis was induced by an atherogenic diet (1.25% cholesterol, 15% fat and 0.5% cholic acid) combined with lipopolysaccharide (LPS), artesunate markedly reduced TC and LDL levels, which was comparable to the effect of rosuvastatin (67,68). Similar lipid-lowering and anti-atherosclerotic effects of artesunate have also been reported in HFD-fed ApoE^−/−^ mice and Western diet-fed rabbits (61,69). This discrepancy may be attributed to the absence of functional ApoE in ApoE^−^/^−^ mice, as ApoE is a required ligand for LDL receptor-mediated lipoprotein clearance, and this genetic deficiency may limit the cholesterol-lowering efficacy of artesunate (61,67). Artemisinin ester may also participate in activating PPARα-related pathways and promoting weight loss and energy expenditure, thereby indirectly improving systemic lipid metabolism (20). Regarding safety, while high doses of artemether may induce gastrointestinal suppression in mice, overall preclinical data indicate no severe adverse effects were observed in this study and the long-term effects of artemether remain uncharacterized as they were not explored in the aforementioned research (21).

Research on NASH models with different etiologies (for example obesity or diabetes mellitus) has not been conducted and clinical translation data are limited (48). Additionally, the regulatory effects of artemether on lipid profiles yield inconsistent results across different animal models and unified efficacy evaluation criteria are lacking (20). Preclinical studies and early-phase clinical trials are needed to test artemisinin-based drugs at low doses over extended periods. These studies should aim to refine the understanding of the cross-regulatory network of the gut-liver axis, standardize research models and evaluation criteria and explore the efficacy of combination therapies with different types of artemisinin derivatives to advance their clinical translation.

### Anti-inflammatory and immune regulatory effects

**Figure 3.**
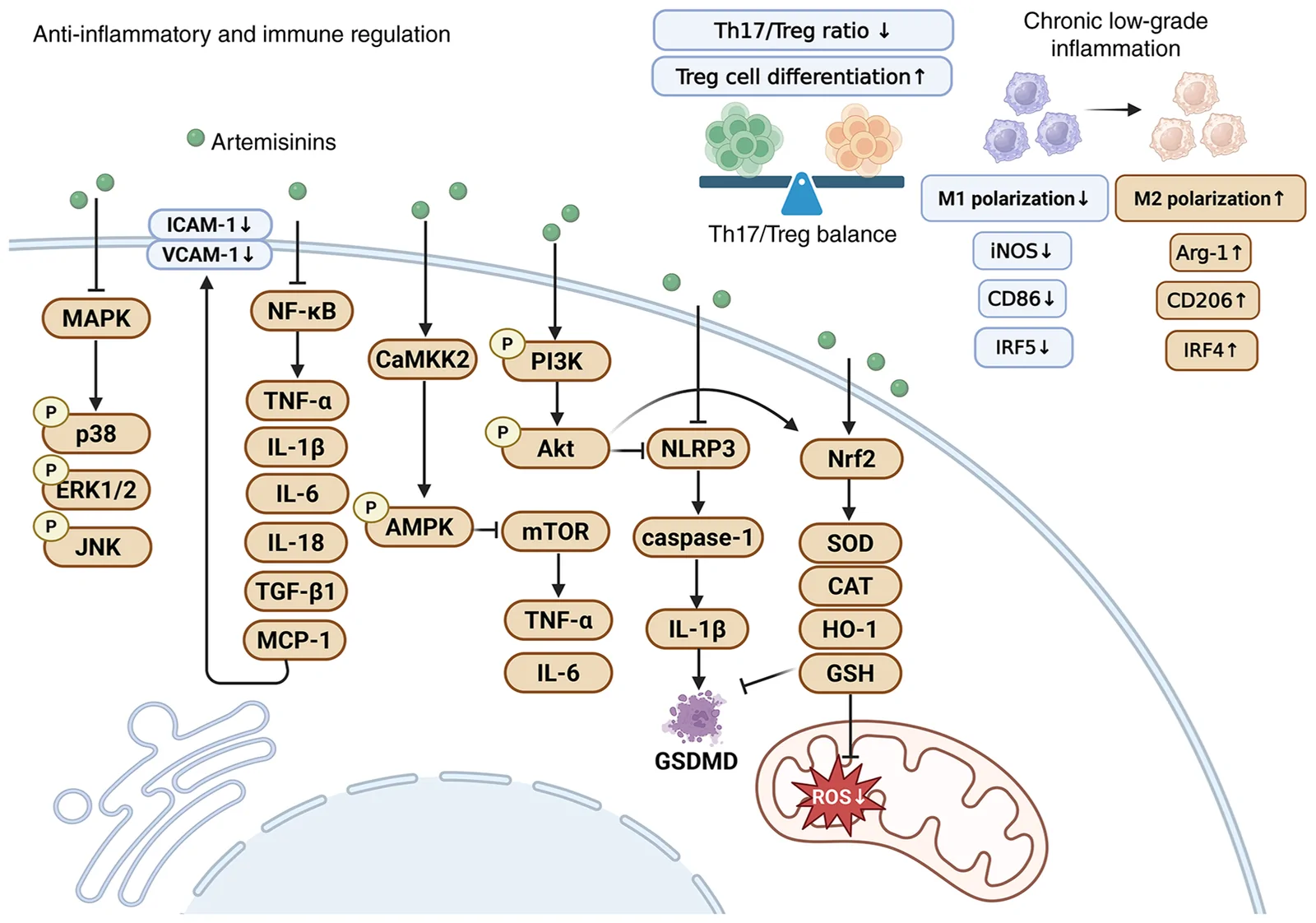
Artemisinin and its derivatives suppress chronic inflammation and restore immune homeostasis through modulation of key signaling pathways and immune cell differentiation. Artemisinin derivatives inhibit MAPK/NF-κB signaling, reducing pro-inflammatory cytokine and adhesion molecule production, while activating PI3K/Akt/mTOR and Nrf2 pathways to reinforce antioxidant defenses. Artemisinin and its derivatives also attenuate NLRP3 inflammasome activation, promote Treg differentiation and drive macrophage polarization from the M1 toward the M2 phenotype. Collectively, these actions alleviate chronic inflammation and help restore immune balance in MetS. Created with BioRender.com. AMPK, AMP-activated protein kinase; Arg-1, arginase 1; CaMKK2, calcium/calmodulin-dependent protein kinase kinase 2; CAT, catalase; GSDMD, gasdermin D; GSH, glutathione; HO-1, heme oxygenase-1; ICAM-1, intercellular adhesion molecule-1; iNOS, inducible nitric oxide synthase; IRF4, interferon regulatory factor 4; IRF5, interferon regulatory factor 5; MetS, metabolic syndrome; MCP-1, monocyte chemoattractant protein-1; NLRP, nucleotide-binding oligomerization domain, leucine-rich repeat and pyrin domain-containing protein; Nrf2, nuclear factor erythroid 2-related factor 2; ROS, reactive oxygen species; SOD, superoxide dismutase; Treg, regulatory T cell; Th17, T helper 17 cell; VCAM-1, vascular cell adhesion molecule-1.

Chronic low-grade inflammation and immune homeostasis imbalance are core pathological features of MetS that are primarily manifested by excessive activation of innate immune cells, dysregulation of adaptive immune responses and an imbalance between anti- and pro-inflammatory reactions. These factors continuously exacerbate insulin resistance and disturbances involving glucose and lipid metabolism, forming a vicious cycle of inflammation-metabolism (70–73). Artemisinin and its derivatives correct the inflammation and immune dysregulation associated with MetS by inhibiting pro-inflammatory pathways, activating antioxidant signaling and regulating immune cell homeostasis (Fig. 3).

### Vascular tissue

At the molecular level, DHA suppresses NF-κB activation by blocking upstream signaling and directly binding to NF-κB. This dual inhibition downregulates pro-inflammatory mediators, including TNF-α, IL-1β, IL-6, IL-18, monocyte chemoattractant protein-1 and TGF-β1, as well as adhesion molecules such as intercellular adhesion molecule-1 and vascular cell adhesion molecule-1. Consequently, monocyte adhesion and migration are attenuated and NLRP3 inflammasome activation is restrained. These actions alleviate the chronic low-grade inflammatory immune response in patients with MetS (22,54,74). Furthermore, in ApoE^−^/^−^ mice fed a HFD, artemether inhibited signaling involving the ERK1/2/NF-κB/IL-1β pathway, reduced the macrophage-like phenotypic transformation of vascular smooth muscle cells and decreased NLRP3 and CD68 expression within plaques. Therefore, artemisinin-derived drugs improve underlying metabolic disorders, protect blood vessels and stabilize plaques, highlighting a potential interventional strategy for cardiovascular complications (61).

### Liver

In an NAFLD model, artemether notably alleviated oxidative stress and inflammatory damage in hepatocytes by activating the Nrf2 antioxidant pathway, thereby promoting Nrf2 nuclear translocation and the expression levels of downstream antioxidant enzymes, including SOD and GSH (25).

### Salivary glands

In a rat model of T2DM, artemether notably reduced inflammatory markers such as caspase-1 and IL-1β in the salivary glands by inhibiting the NF-κB/NLRP3 pathway, improving oxidative stress and regulating oral dysbiosis. This indicates that the anti-inflammatory effects of artemether are not limited to traditional metabolic organs but can also improve systemic multi-tissue microinflammation (74).

### Systemic metabolic tissues

In db/db mice, artemisinin effectively alleviated the inflammatory response associated with glucose and lipid metabolism disorders by inhibiting the MAPK signaling pathway, reducing the phosphorylation of p38, ERK1/2 and JNK and activating the PI3K/Akt pathway (48).

### Immune system

At the cellular immune level, artemisinin-based drugs correct metabolic disorder-induced immune imbalances by reshaping the phenotypic and functional balance of immune cells and regulating the functions of key cells in innate and adaptive immunity. DHA selectively regulates adaptive immune cells by promoting CD4^+^ T cell proliferation and enhancing IFN-γ^+^ CD8^+^ T cell activity, while inhibiting germinal center B cell expansion and reducing the number of circulating plasma cells and antibody production. Therefore, DHA can coordinate immune activation and immune tolerance, thereby preventing excessive immune responses from exacerbating metabolic damage and maintaining immune homeostasis (75). Furthermore, the artemisinin-derived compound β-aminoartemether maleate (SM934) can balance the Th17/Treg cell ratio, promote Treg differentiation and inhibit excessive Th17 cell activation, alleviating chronic low-grade inflammation mediated by immune imbalance (41). Regarding the regulation of innate immune cells, artemether upregulates interferon regulatory factor 4 (IRF4) expression in rodent and non-human primate models, promoting the polarization of macrophages toward the anti-inflammatory M2 phenotype and inhibiting conversion to the pro-inflammatory M1 phenotype. This M2-biased polarization promotes IL-10 and TGF-β secretion to exert its anti-inflammatory and tissue-repairing effects while reducing the release of pro-inflammatory factors, such as TNF-α and IL-1β, alleviating the inflammatory response in vascular restenosis. These observations suggest that artemisinin is not a fixed anti- or pro-inflammatory agent; artemisinin is an immunomodulator capable of dynamically adapting to the tissue microenvironment and pathological state (76).

### Non-MetS disease models

By contrast, in patients with glioblastoma and a number of tumor models (including models of breast, lung and cervical cancer *in vitro*, as well as an *in vivo* breast cancer model), artesunate binds to heme to generate ROS, reversing tumor-associated macrophages from an M2-like, tumor-promoting state toward an M1-like, pro-inflammatory and antitumor state. This phenotypic shift reshapes the immune microenvironment and potentiates antitumor immunity (77,78). In the context of malaria infection, DHA promotes macrophage polarization toward the M1 phenotype by activating NOD-like receptor family pyrin domain-containing 12 (NLRP12), thereby enhancing the host's ability to combat *Plasmodium* parasites and facilitating parasite clearance (79). Collectively, these observations demonstrate that the immunomodulatory effects of artemisinin derivatives on macrophage polarization are context-dependent. Rather than exerting a fixed anti- or pro-inflammatory effect, artemisinin derivatives function as immunomodulators that differentially regulate macrophage phenotypes depending on the pathological setting, tissue microenvironment and disease context.

However, the poor water solubility and short half-lives of artemisinin derivatives make it difficult to maintain sustained effects for chronic inflammation (80,81). Furthermore, the immunomodulatory effects of artemisinin-based drugs are dose- and microenvironment-dependent. In a malaria model, DHA induced M1 macrophage polarization via an NLRP12-dependent mechanism yet promoted M2 polarization in metabolic inflammation; the critical threshold for this macrophage phenotypic switch remains undefined (79,82). Future studies should focus on developing long-acting sustained-release or targeted delivery systems to meet the needs of chronic interventions. For instance, apigenin-loaded nanoparticles nanocarriers may enhance the macrophage targeting and M2 polarization regulation effects of artemisinin-based drugs (83).

### Regulation of the gut microbiota-metabolism axis

**Figure 4.**
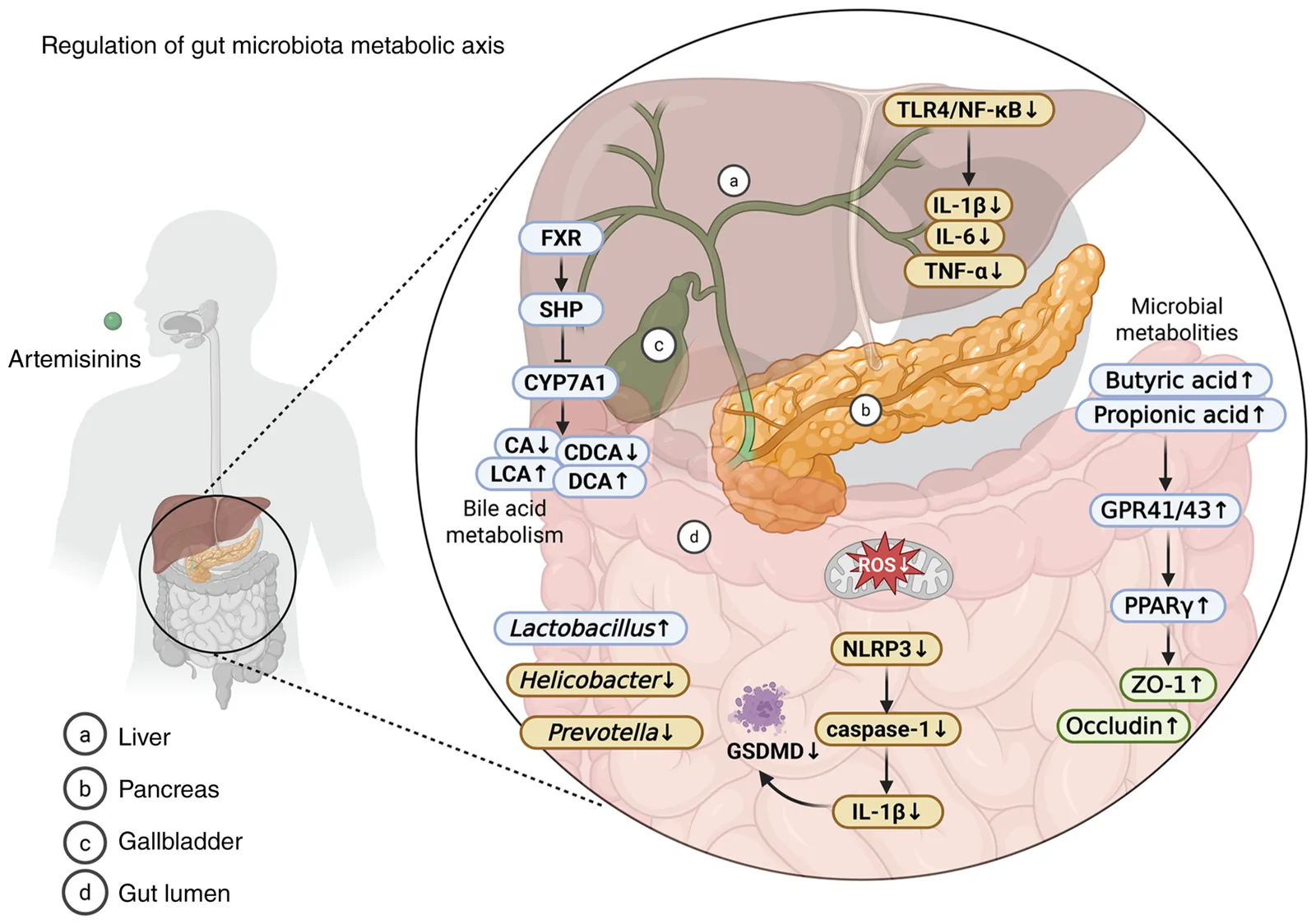
Artemisinin and its derivatives preserve intestinal barrier integrity by upregulating tight junction proteins (ZO-1 and occludin) and boosting short-chain fatty acids (butyrate and propionate) via GPR41/43 and PPARγ, while regulating FXR/CYP7A1-mediated bile acid metabolism and restraining endotoxin translocation to protect hepatic function through the gut-liver axis. Notably, gut microbiota-related pathways in this schematic are merely correlational, lacking rigorous causal validation. Created with BioRender.com. CA, cholic acid; CDCA, chenodeoxycholic acid; CYP7A1, cholesterol 7α-hydroxylase; DCA, deoxycholic acid; FXR, farnesoid X receptor; GPR41/43, G protein-coupled receptor 41/43; GSDMD, gasdermin D; LCA, lithocholic acid; NLRP, nucleotide-binding oligomerization domain, leucine-rich repeat and pyrin domain-containing protein; PPARγ, peroxisome proliferator-activated receptor γ; ROS, reactive oxygen species; SCFA, short-chain fatty acid; SHP, small heterodimer partner; ZO-1, zonula occludens-1.

Dysregulation of the gut microbiota-metabolism axis is a key pathogenic mechanism underlying MetS. The gut-microbiota-metabolism axis mediates critical processes such as bile acid reprogramming and tryptophan metabolism through bi-directional pathways, including gut-liver and gut-spleen interactions. Disruption of the gut-microbiota-metabolism axis leads to abnormal metabolites, including secondary bile acids, hydrogen sulfide and dysregulated short-chain fatty acids, which are transmitted via the gut-systemic metabolic axis, severely affecting the host's metabolic and immune balance (84–88). Artemisinin and its derivatives have been reported to modulate this axis, with effects associated with microbiota-dependent pathways. By reshaping the gut microbiota structure, artemisinin and its derivatives optimize the profile of microbial metabolites, regulate the bile acid signaling axis, strengthen the barrier function of the gut-liver axis, alleviate intestinal inflammation and ultimately contribute to the restoration of gut microbiota homeostasis (Fig. 4).

### Intestine

One animal study has shown that artemisinin derivatives are associated with alterations in gut microbiota composition. DHA enhances the α-diversity of the gut microbiota and reshapes the microbial ecosystem structure in mice without causing pathological damage to the liver, kidneys or intestines. DHA increases the abundance of Firmicutes and Spirochaetes while reducing the proportion of Bacteroidetes and Actinobacteria, and these compositional shifts are accompanied by anti-inflammatory and metabolic regulatory effects at the functional level. Metabolic analysis has shown that DHA reduces serum TG levels, with TG content exhibiting a positive correlation with the abundance of Deferribacterota and a negative correlation with the abundance of *Spirochaeta*, suggesting that the lipid-regulating effects of DHA are partially dependent on gut microbiota remodeling (89). Conversely, artemether increases the abundance of *Lactobacillus* while reducing the abundance of pathogenic bacteria such as *Helicobacter* and *Prevotella*. These changes are associated with AMPK activation, inhibition of the mTOR/NF-κB pathway, reduced pro-inflammatory factor levels and improved insulin resistance and liver and pancreatic tissue damage, suggesting a potential ‘microbiota-metabolism-immunity’ axis that may contribute to the effects of artemisinin-based drugs in the prevention and treatment of T2DM (90). At the molecular level, several pathways have been proposed to link artemisinin-based drugs to the gut microbiota-metabolism axis, with the tryptophan metabolic pathway being among the most studied. Artemether can inhibit the tryptophan-kynurenine metabolic pathway via a heme-dependent mechanism, blocking indoleamine 2,3-dioxygenase 1 (IDO1) activity and reducing kynurenine production. By binding to the heme-binding pocket of IDO1 to inhibit enzyme activity, artemether alleviates kynurenine-mediated inflammation and improves the redox balance of intestinal cells (91). DHA increases the abundance of butyrate-producing bacteria and butyrate levels, whereas artemether increases the content of short-chain fatty acids such as propionate and valerate. One potential mechanism is that these short-chain fatty acids activate G protein-coupled receptor 41/43 (GPR41/43) receptors and the PPARγ pathway, which in turn enhances the expression levels of tight junction proteins, such as zonula occludens-1 and occludin, in the colon and reduces intestinal permeability. This could prevent endotoxins (for example LPS) from entering the bloodstream via the gut and if so, might suppress chronic low-grade inflammation mediated by the gut-metabolic axis and thereby improve insulin resistance (40,90,92).

### Liver

Within the framework of the gut microbiota-metabolite axis, the gut-liver axis acts as an important signaling conduit that transmits microbiota-derived metabolic signals to the liver, and represents a plausible route through which artemisinin and its derivatives exert hepatoprotective effects. In terms of liver protection, DHA, as a farnesoid X receptor (FXR) agonist, downregulates the expression level of cholesterol 7α-hydroxylase (CYP7A1), which is a key rate-limiting enzyme in the conversion of cholesterol to bile acids, via FXR activation. Through this mechanism, DHA could mitigate the liver damage caused by excessive bile acid accumulation, alleviate hepatic steatosis and holistically protect liver function while controlling obesity (93–97). Together, these pathways could conceivably constitute a microbiota-metabolite-systemic immunity tripartite regulatory network, enabling artemisinin-based drugs to modulate the gut microbiota-metabolite axis.

### Spleen

Circulating metabolites originating from gut microbiota can mediate systemic immune regulation, affecting peripheral immune organs such as the spleen. At the immune regulatory level, artemether can inhibit the activation of pro-inflammatory macrophages, modulate the splenic immune microenvironment and immune cell function and restore host immune homeostasis (98).

Notably, the microbiota-related mechanisms discussed are predominantly correlational and the specific contributions of gut microbiota remodeling to the metabolic effects of artemisinin derivatives remain to be causally established. Causal evidence for microbiota dependence remains tenuous, as direct validation using germ-free animals, antibiotic depletion studies or fecal microbiota transplantation have not yet been reported. Clinical translation is still in its infancy, and the relative merits of distinct artemisinin derivatives in modulating the gut-liver axis remain unclear (99). Future research should prioritize exploratory clinical trials built on the microbiota-metabolite-immune axis to evaluate the long-term impacts of treatment on gut microbiome stability.

### Evidence grading and mechanism validation summary

#### Evidence stratification criteria

Evidence is tiered according to the type of experimental disease models employed. Direct metabolic disease models, namely db/db mice, T2DM rodents and HFD-induced obese mice, generate the most robust evidence to validate the metabolic regulatory functions of artemisinin derivatives. Data obtained from NAFLD/NASH and atherosclerosis models offer moderate supporting evidence; these disorders share partial pathological features with MetS yet come with intrinsic limitations due to incomplete pathological overlap. Mechanistic clues derived from cancer and malaria models remain indirect and preliminary, and further validation in metabolic animal models is mandatory before definitive conclusions related to MetS treatment can be established.

### Reliability grading of functional pathways

Multiple pathways governing the metabolic effects of artemisinin derivatives are fully validated in db/db mice, HFD-induced obesity models and T2DM animals. The hepatic-skeletal muscle PI3K/Akt-GLUT2/GLUT4 axis optimizes glucose metabolism and suppresses gluconeogenesis, as demonstrated in db/db mice and HFD-induced obesity models (48,100,101). AMPK/Nrf2 alleviates oxidative stress-induced insulin resistance, the MAPK/NF-κB pathway breaks the inflammation-insulin resistance cycle and IRF4 reduces systemic inflammation by inducing M2 macrophage polarization, with these mechanisms supported by studies in human retinal pigment epithelial cells, bronchial epithelial cells, Nrf2-knockdown experiments, and rodent and non-human primate models of vascular restenosis (55,56,76). Collectively, these findings constitute direct evidence, as they are derived from both metabolic disease models and mechanistic loss-of-function studies, thereby supporting the classification of these pathways as well-supported.

A second set of pathways are supported by moderate evidence, characterized only in NAFLD, ApoE^−^/^−^ atherosclerotic and other analogous models without systematic verification in MetS, resulting in inconsistent efficacy across species. Hepatic AMPK-SREBP-1c inhibits lipogenesis with variable lipid-lowering effects, as observed in methionine- and choline-deficient (MCD) diet-fed and HFD-fed murine NASH models (60). The gut-liver FXR/CYP7A1 pathway improves hepatic steatosis but lacks long-term validation in obese MetS models, with evidence derived primarily from studies using hepatocellular carcinoma cells and conventional rodent models (93–97). Vascular KLF2/Nrf2/TCF7L2 prevents atherosclerosis with unconfirmed hypoglycemic activity, as demonstrated in ApoE^−^/^−^ mice fed a HFD (22). Hepatic TLR4/NF-κB suppresses inflammation in Kupffer cells within NASH models, yet its link to gut microbiota remains unclear, and PPARα/CPT1 combined with EGFR/HSP90 alleviates fatty liver with poor experimental reproducibility, based on findings in MCD diet-fed mice and oleic acid/palmitic acid-treated HepG2 cells (60,62–64).

The remaining pathways are supported only by indirect speculative evidence, among which certain mechanisms are summarized from malaria and tumor research. Most intestinal-related mechanisms are also grouped in the present review, including the microbiota-metabolism-immune network, the tryptophan-kynurenine-IDO1 cascade and the SCFA-GPR41/43-PPARγ intestinal barrier axis, as these are backed solely by correlational results without causal validation using germ-free animals or fecal microbiota transplantation (89–92). Moreover, the inverse regulation of PI3K/Akt/mTOR detected in melanoma cells highlights the context-dependent pharmacological properties of artemisinin derivatives, indicating that findings from non-metabolic disease models require further validation before extrapolation to metabolic tissue (57).

## 4. Clinical applications and emerging therapeutic strategies for artemisinin and its derivatives

Artemisinin and its derivatives have a well-established role in malaria treatment, but their translation to MetS requires a distinct clinical framework focused on metabolic and inflammatory endpoints.

### Candidate derivatives and formulation optimization

Among artemisinin derivatives, SM934 represents not only a formulation-optimized derivative with improved aqueous solubility but is also a candidate with preclinical evidence in MetS. In HFD-induced obese mice, SM934 reduced body weight gain, improved glucose tolerance and insulin sensitivity and decreased ectopic lipid deposition in the liver. Mechanistically, SM934 inhibits α-enolase to lower phosphoenolpyruvate (PEP). PEP stabilizes transforming growth factor-β-activated kinase 1 to drive NF-κB-dependent inflammation, SM934 blocks this cascade, curbing macrophage IL-1β secretion and lowering the adipose M1/M2 ratio to relieve obesity-linked meta-inflammation (102). Meanwhile, SM934 activates Nrf2 and represses NF-κB/NLRP3 inflammasome signaling to restore overall immune homeostasis (103). Phase I trials have supported the favorable tolerability profile of SM934 in healthy participants (registered under CTR20160932 and CTR20160933 in the Drug Clinical Trial Registration and Information Disclosure Platform; available a www.chinadrugtrials.org.cn), and a Phase II trial in patients with systemic lupus erythematosus is currently underway (41). In addition, SM934 has demonstrated notable efficacy in various autoimmune and inflammatory disease models, including systemic lupus erythematosus, rheumatoid arthritis, inflammatory bowel disease, membranous nephropathy and dry eye syndrome (41).

### Target patient phenotypes and clinical endpoints

MetS encompasses a heterogeneous patient population and clinical development should prioritize well-defined subgroups with unmet needs. Polyendocrine metabolic ovarian syndrome (PMOS) represents one of the most advanced clinical targets. A single-arm pilot study in 19 patients with PMOS demonstrated that 40 mg DHA three times daily for 12 weeks normalized menstrual cycles in 63.16% of participants, while also lowering total testosterone and reducing antral follicle count (104). Metabolic dysfunction-associated fatty liver disease (MAFLD) is another priority target. A proof-of-concept trial (NCT07679542) is assessing whether DHA (20 mg three times daily for 12 weeks) reduces liver fat in adults with MAFLD, using MRI-proton density fat fraction-assessed hepatic fat content as the primary endpoint (105). These ongoing proof-of-concept studies in defined patient cohorts represent the initial steps toward a dedicated clinical development pathway for MetS. Candidate clinical endpoints for MetS interventions should include enhanced insulin sensitivity, as assessed by the homeostatic model assessment of insulin resistance, improved glycemic control quantified by glycated hemoglobin A1c, favorable lipid modifications (TG, LDL-C and HDL-C), decreased inflammatory markers (IL-6 and TNF-α) and ultimately, lowered cardiovascular event risk or arrested T2DM progression.

### Dosing, duration and long-term safety

Chronic MetS management requires different dosing strategies from acute malaria treatment. Evidence from a preclinical study suggests that long-term drug exposure, rather than short-term peak concentrations, is the primary driver of artemisinin-related toxicity (106). Rapid oral elimination is therefore associated with a more favorable safety profile compared with delayed release from intramuscular formulations (107). Nevertheless, a 15-year retrospective analysis of artemether-related adverse events from the FDA Adverse Event Reporting System identified hemolytic anemia and hemolysis as the most frequently reported events, with women showing a higher incidence of reproductive-related events, including spontaneous abortion and premature labor. Most adverse events occurred within the first 30 days of artemether administration, with the most affected systems being the hematologic and hepatobiliary systems (108). To date, most clinical trials of artemisinin derivatives have been limited to small-sample Phase I malaria studies. Larger-scale Phase II/III trials are therefore needed to comprehensively evaluate both efficacy and long-term safety in metabolic populations. These findings underscore the need for systematic long-term safety monitoring in future MetS trials, including hematological, hepatic and reproductive assessments.

### Drug-drug interactions with standard MetS medications

Patients with MetS frequently receive multiple concomitant medications, including metformin, statins, SGLT2 inhibitors, glucagon-like peptide-1 (GLP-1) receptor agonists and antihypertensives. Artemisinin and its derivatives are primarily metabolized by cytochrome P450 2B6 (CYP2B6) and cytochrome P450 3A (CYP3A). Artemisinin can induce CYP2B6 and CYP3A4 protein expression, raising the potential for pharmacokinetic interactions with co-administered drugs metabolized by these pathways (109). Reduced enzyme function may lead to subtherapeutic concentrations of the active metabolite DHA, potentially compromising treatment efficacy. Interactions with cardiovascular, antibiotic and antiparasitic agents have been reported, and clinicians should remain vigilant for potential drug-drug interactions when repurposing these agents for chronic metabolic indications (110). Systematic evaluation of interactions with standard MetS medications (including metformin, statins, SGLT2 inhibitors and GLP-1 receptor agonists) are therefore essential before clinical adoption, with careful attention to patient stratification and drug-drug interaction monitoring.

### Antimalarial resistance risks

Expanding artemisinin use for non-malarial indications raises legitimate concerns about accelerating antimalarial resistance. Evidence suggests that prolonged or widespread use in malaria-endemic regions could exert selection pressure on *Plasmodium* parasites (33). To mitigate this risk, non-antimalarial derivatives with altered metabolic profiles, such as SM934, which exhibits distinct immunosuppressive mechanisms independent of the antimalarial activity of SM934, or formulations with shorter exposure windows should be prioritized for MetS indications. Additionally, combination regimens analogous to triple artemisinin combination therapies could be considered to reduce resistance selection pressure in chronic use settings (111). These efforts, combined with robust resistance surveillance, will determine whether artemisinin derivatives can successfully transition from antimalarial agents to viable therapeutics for MetS.

## 5. Conclusion and outlook

The global prevalence of MetS shows a sustained and rapid increasing trend. Between 2000 and 2023, MetS prevalence between women and men globally rose from 14.7 to 31.0% and 9.0 to 25.7%, respectively. As of 2023, ~1.54 billion adults worldwide were affected by MetS (112). This condition increases the risk of T2DM, cardiovascular disease, NAFLD and various types of cancer and poses a severe challenge to global public health (113,114). Given the complex nature of MetS, artemisinin and its derivatives have attracted research attention as potential preclinical candidates, largely due to their ability to affect multiple disease-related pathways. Their mechanisms include improving insulin resistance, regulating glucose and lipid metabolism, alleviating chronic inflammation and restoring gut microbiota homeostasis. These effects involve signaling pathways including molecules, such as AMPK, PI3K/Akt, NF-κB and MAPK, and gene regulation, such as SREBF1, PPARA, SLC2A2 and IRS1. Therefore, downregulating the expression levels of proteins such as TNF-α, IL-1β, IL-6 and caspase-1, upregulating IL-10 and TGF-β, and regulating macrophage M1/M2 polarization, Th17/Treg differentiation, tryptophan metabolism, short-chain fatty acid production and the bile acid FXR/Takeda G protein-coupled receptor 5 signaling axis is key. Tables I and II summarize the roles of artemisinin and its derivatives in MetS-related models (Table I presents direct evidence derived from MetS-related models, whereas Table II summarizes indirect mechanistic evidence obtained from non-MetS models).

Although artemisinin and its derivatives have demonstrated potential in the treatment of MetS, several limitations remain. First, regarding low bioavailability, artemisinin-based drugs generally have poor water solubility and variable oral absorption, leading to insufficient systemic exposure and hindering their full therapeutic potential. Second, chronic dosing regimens for MetS remain uncertain. Unlike the short-course, high-dose regimens used for malaria treatment, MetS would require long-term, sustained administration. The appropriate dose, frequency and duration of treatment have not been established, and the pharmacokinetic-pharmacodynamic relationships under chronic dosing conditions remain poorly understood. Third, there is a lack of high-quality, MetS-specific clinical trials, with most studies focusing on the cellular and animal experimental stages, whereas clinical research consists primarily of single-arm exploratory trials with small samples, and large-scale, controlled clinical trials are lacking. Such trials would be needed to validate the long-term efficacy and safety of artemisinin-based drugs. Fourth, long-term safety profiles remain unclear. Studies have largely focused on short-term efficacy observations and systematic evaluations of potential toxicity. Delayed adverse reactions following repeated use or long-term exposure have not been conducted. Furthermore, in various animal studies, the therapeutic effects of artemisinin and its derivatives vary with dose. Relatively low doses may not produce toxicity, which is crucial for determining a safe and effective dosage range. Fifth, possible drug-drug interactions represent an understudied concern. Artemisinin derivatives are known to modulate cytochrome P450 enzymes, which could alter the metabolism of commonly prescribed MetS medications, including statins, metformin and antihypertensive agents. However, mechanistic and clinical studies on such interactions remain scarce and the associated risks are unclear. Sixth, evidence for specific populations is lacking, with a severe shortage of pharmacokinetic, efficacy and safety data in vulnerable populations, such as young children and pregnant women, limiting the widespread clinical application of these drugs. Finally, the public health issue of antimalarial resistance cannot be overlooked. While artemisinin-based combination therapies remain the mainstay of malaria treatment, partial resistance has emerged across Africa and Southeast Asia (115). The long-term use of these drugs for MetS could accelerate the spread of resistance, necessitating careful risk assessment and consideration of non-antimalarial derivatives or formulations with lower antimalarial selection pressure. Notably, unlike conventional artemisinins that are metabolized to DHA, a process linked to parasite quiescence induction and resistance development, non-antimalarial analogues such as 11-aza-artemisinins and the artemisone/artemiside series do not generate DHA, yet remain potent against multidrug-resistant strains, with no detectable artemisone resistance and even greater efficacy for artemiside, supporting their prioritization for metabolic indications (116,117).

In summary, non-clinical research surrounding artemisinin and its derivatives has made progress; however, several issues and limitations remain. Future efforts should focus on addressing these challenges through in-depth research to elucidate the unclear molecular mechanisms, establishing the basis for rational drug design. This approach should be combined with long-term clinical trials to establish safety parameters for long-term use. Concurrently, efforts should be directed toward developing safe and efficient drug delivery systems and exploring novel formulations to enhance bioavailability and target specificity. This approach will facilitate the clinical translation of artemisinin and its derivatives to interventions for human disease.

## Figures and Tables

**Table I.** Roles of artemisinin and its derivatives in MetS-related models: Direct evidence.

First author/s, year	Compound	Disease model	Species/cell type	Dose, route and duration	Endpoint (metabolic/inflammatory)	Proposed mechanism	Evidence level	Limitation	(Refs.)
Guo *et al*, 2024	Artesunate	Obesity	Male C57BL/6J mice (HFD-induced obese, DIO)	5 or 20 mg/kg, i.p., once every 2 days, 13–60 days	Metabolic: Body weight ↓; food intake ↓; glucolipid metabolism ↑; insulin sensitivity ↑.	GDF15 ↑; CHOP ↑; c-Fos^+^ ↑	Strong	Only male mice tested; immune endpoints not evaluated	(20)
					Inflammatory: Not assessed				
Wu *et al*, 2022	Artemisinin	T2DM	SD rats; Wistar rats; db/db mice	25-300 mg/kg/day, i.g. or i.p., 4–12 weeks	Metabolic: Glycemic control ↑; insulin secretion ↑; oxidative stress ↓.	Arx ↓; Nrf2 ↑; TNF-α/IL-6/MCP-1 ↓	Strong	α-to-β cell trans-differentiation remains	(21)
					Inflammatory:			controversial	
					Pro-inflammatory cytokines ↓				
Wu *et al*, 2022	Artesunate	T2DM	SD rats; Wistar rats; db/db mice	10-100 mg/kg/day, i.g. or i.p., 4–12 weeks	Metabolic: Blood glucose ↓; pancreatic β-cell function ↑. Inflammatory: Inflammatory signaling ↓	SIRT1 ↑; NF-κB ↓; BCL-2/BCL-X ↓, BAX ↑	Strong	Broad dose range; optimal dose not established	(21)
Wu *et al*, 2022	Artemether	T2DM	db/db mice	100-400 mg/kg/day, i.g., 4 weeks	Metabolic: Fasting blood glucose ↓; glucolipid metabolism ↑.	AMPK ↑; GLUT4/IRS1 ↑	Strong	Immune endpoints not evaluated	(21)
					Inflammatory: Not assessed				
Majidiani *et al*, 2025	Artemisinin	Athero-sclerosis	ApoE^−^/^−^ mice, HFD	50 or 100 mg/kg, i.g., 8 weeks	Metabolic: Serum lipid profile improvement. Inflammatory: Macrophage inflammation ↓	AMPK ↑; mTOR ↓; ULK1 ↓; p62 ↓; LC3-II ↑; NF-κB ↓; NLRP3 ↓; IL-1β/IL-18/TGF-β1 ↓	Moderate	ApoE^−^/^−^ background may limit translation to normal lipid metabolism	(22)
Chen *et al*, 2024	Artesunate	T2DM	C57BL/KsJ-db/db mice	160 mg/kg/day, i.g., 7 weeks	Metabolic: Fasting glucose ↓; fasting insulin ↓; insulin resistance ↓; serum lipids ↑; hepatic steatosis ↓. Inflammatory: Not directly assessed	PI3K/Akt ↑; MAPK ↓	Strong	Single dose tested; no inflammation-specific endpoint reported	(48)
Xu *et al*, 2022	Artemether	NASH	*In vivo*: C57BL/6J mice (MCD diet 4 week + HFD 28 week) *In vitro*: HepG2 cells treated with oleic acid and palmitic acid	*In vivo*: MCD: 100 or 200 mg/kg/day, i.g., 2 week; HFD: 20 mg/kg/week, i.p., 7 week; *In vitro*: 6.25 or 25 µM, 24 h	Metabolic: Serum transaminases ↓; hepatic steatosis ↓. Inflammatory: Hepatic inflammation ↓; pro-inflammatory cytokines ↓; IL-10 ↑	SREBP-1c/ACC/FASN/SCD1 ↓; ATGL/PGC-1α/CPT-1a ↑; NF-κB/TNF-α/IL-1β/M1 ↓; IL-10 ↑ TGF-β/α-SMA/Smad2 ↓	Moderate	MCD diet model may not fully recapitulate human NASH; no clinical validation	(60)
Liu *et al*, 2024	Artesunate	Athero-sclerosis	*In vivo*: ApoE^−^/^−^mice, HFD; *In vitro*: HVSMCs	*In vivo*: 10 mg/kg/day, i.p., 12 week; *In vitro*: 50–100 µM	Metabolic: Serum lipids ↑ (TG, TC, VLDL-C, LDL-C ↓). Inflammatory: Plaque inflammation ↓; inflammasome activation ↓	NLRP3/caspase-1/IL-1β ↓; MAPK/NF-κB/AP-1 ↓; HPSE/MMP9/KLF4 ↓	Moderate	ApoE^−^/^−^ background; no systemic inflammatory markers assessed	(61)
Jiang *et al*, 2025	Artemether	T2DM	C57BL/KsJ-db/db mice	80 or 160 mg/kg/day, i.g., 8 weeks	Metabolic: Blood glucose ↑; insulin sensitivity ↑; serum lipids ↑; liver/pancreas pathology ↑. Inflammatory: Inflammatory response ↓; NF-κB signaling ↓	AMPK ↑; mTOR ↓; p38 MAPK ↓; NF-κB ↓; IκBα ↓; IRS1 ↓; TLR4 ↓; Occludin ↑; ZO-1/Claudin-1 ↑	Strong	Only one resistant model; no human data	(90)

↑, increase or upregulation; ↓, decrease or downregulation; ACC, acetyl-CoA carboxylase; AMPK, adenosine monophosphate-activated protein kinase; ApoE^−^/^−^, apolipoprotein E knockout; Arx, aristaless-related homeobox; ATGL, adipose triglyceride lipase; c-Fos, FBJ murine osteosarcoma viral oncogene homolog; CHOP, C/EBP homologous protein; CPT-1a, carnitine palmitoyltransferase 1a; DIO, diet-induced obesity; FASN, fatty acid synthase; GDF15, growth differentiation factor 15; GLUT4, glucose transporter type 4; HDL-C, high-density lipoprotein cholesterol; HFD, high-fat diet; HPSE, heparanase; HVSMC, human vascular smooth muscle cell; IκBα, NF-κB inhibitor α; i.g., intragastric; i.p., intraperitoneal; IRS1, insulin receptor substrate 1; KLF4, Krüppel-like factor 4; LC3-II, microtubule-associated protein 1 light chain 3-II; LDL-C, low-density lipoprotein cholesterol; MCD, methionine- and choline-deficient diet; MCP-1, monocyte chemoattractant protein-1; NASH, non-alcoholic steatohepatitis; NLRP, nucleotide-binding oligomerization domain, leucine-rich repeat and pyrin domain-containing protein; Nrf2, nuclear factor erythroid 2-related factor 2; PGC-1α, peroxisome proliferator-activated receptor γ coactivator 1-α; SCD1, stearoyl-CoA desaturase 1; SD, Sprague-Dawley; SIRT1, sirtuin 1; SMA, smooth muscle actin; SREBP-1c, sterol regulatory element-binding protein 1c; T2DM, type 2 diabetes mellitus; TC, total cholesterol; TG, triglyceride; TLR4, Toll-like receptor 4; ULK1, Unc-51 like autophagy activating kinase 1; VLDL-C, very low-density lipoprotein cholesterol; ZO-1, zonula occludens-1.

**Table II.** Roles of artemisinin and its derivatives in non-MetS models: Indirect mechanistic evidence.

First author/s, year	Compound	Disease model	Species/cell type	Dose, route and duration	Endpoint (metabolic/inflammatory)	Proposed mechanism	Evidence level	Limitation	(Refs.)
Long *et al*, 2024	DC32 (DHA derivative)	Rheumatoid arthritis	DBA/1 mice, collagen-induced arthritis model	25 mg/kg, oral, 30 days	Metabolic: Not assessed. Inflammatory: Joint inflammation ↓, cartilage/bone destruction ↓, Treg/Th17 balance restored	Nrf2/HO-1 ↑; p62 ↑; Keap1 ↓; RORγt ↓; Foxp3 ↑; IL-6/IL-17 ↓	Indirect	Mechanistically suggestive but not in MetS context; no metabolic endpoints evaluated	(54)
Li *et al*, 2022	DHA	Malaria	BALB/c or C57BL/6 mice infected with *P. berghei* ANKA	200 µl/day of 0.1 mg/ml DHA solution, i.g., intermittent preventive treatment	Metabolic: Not assessed. Inflammatory: Anti-malarial cellular immunity ↑; peak parasitemia delayed	MAPK ↑; CD25 ↑; Tim-3 ↓	Indirect	Malaria model not relevant to MetS; no metabolic endpoints evaluated	(75)
Li *et al*, 2025	DHA	Malaria	*In vitro*: RAW264.7 macrophages + iRBC *In vivo*: C57BL/6 mice infected with *P. berghei* ANKA	*In vitro*: 700 µM DHA (co-incubated with iRBC) *In vivo*: 2 mg/mouse oral DHA, 2 days starting 12 h post-infection	Metabolic: Not assessed. Inflammatory: M1 macrophage polarization ↑, parasite clearance ↑	NLRP12 ↑; M1 ↑ (CD86/i NOS/IRF5); M2 ↓ (Arg1/CD206/IRF4); IL-12/IL-6/MCP-1/TNF-α ↑; TSPO ↓; Lcn2 ↑	Indirect	Opposite macrophage polarization direction compared with metabolic contexts; MetS relevance unclear	(79)
Guo *et al*, 2022	DHA	Hepatocellular carcinoma	*In vitro*: HepG2, HepG2215 cells *In vivo*: C57BL/6 mice (DEN/TCPOBOP-induced HCC model)	*In vitro*: 21.5 µM, 24 h; *In vivo*: 25 mg/kg, i.p.	Metabolic: Liver histopathology ↑; HCC proliferation ↓ Inflammatory: Not assessed	FXR ↑; YAP1 ↓; CYP7A1 ↓; NR5A2 (LRH-1) ↓; CYP27A1 ↓; CA ↓; CDCA ↓	Indirect	Cancer model not MetS-relevant; FXR activation may be context-dependent	(93)

↑, increase or upregulation; ↓, decrease or downregulation; Arg-1, arginase 1; CA, cholic acid; CDCA, chenodeoxycholic acid; CYP27A1, cytochrome P450 27A1; DHA, dihydroartemisinin; DEN, diethylnitrosamine; Foxp3, forkhead box P3; FXR, farnesoid X receptor; HO-1, heme oxygenase-1; i.g., intragastric; iNOS, inducible nitric oxide synthase; i.p., intraperitoneal; iRBC, infected red blood cells; IRF4, interferon regulatory factor 4; IRF5, interferon regulatory factor 5; Keap1, Kelch-like ECH-associated protein 1; Lcn2, lipocalin 2; LRH-1, liver receptor homolog-1; MCP-1, monocyte chemoattractant protein-1; MetS, metabolic syndrome; NLRP, nucleotide-binding oligomerization domain, leucine-rich repeat and pyrin domain-containing protein; NR5A2, nuclear receptor subfamily 5 group A member 2; Nrf2, nuclear factor erythroid 2-related factor 2; *P. berghei* ANKA, *Plasmodium berghei* ANKA strain; RORγt, retinoic acid receptor-related orphan receptor γ t; TCPOBOP, 1,4-bis[2-(3,5-dichloropyridyloxy)]benzene; Th17, T helper 17 cell; Tim-3, T cell immunoglobulin and mucin domain-containing protein 3; Treg, regulatory T cell; TSPO, translocator protein; YAP1, Yes-associated protein 1.

## Data Availability

Not applicable.

## Abbreviations

Acaca: acetyl-CoA carboxylase α
AMPK: AMP-activated protein kinase
ApoE^−^/^−^: apolipoprotein E knockout
CPT1: carnitine palmitoyltransferase 1
CYP2B6: cytochrome P450 2B6
CYP3A: cytochrome P450 3A
CYP7A1: cholesterol 7α-hydroxylase
DHA: dihydroartemisinin
FASN: fatty acid synthase
FDA: Food and Drug Administration
FXR: farnesoid X receptor
GLP-1: glucagon-like peptide-1
GLUT: glucose transporter
GPR41/43: G protein-coupled receptor 41/43
GSH: glutathione
HDL-C: high density lipoprotein cholesterol
HSP90: heat shock protein 90
MCD: methionine- and choline-deficient
IDO1: indoleamine 2,3-dioxygenase 1
IRF4: interferon regulatory factor 4
IRS1: insulin receptor substrate 1
KLF2: Krüppel-like factor 2
LDL: low-density lipoprotein
LDL-C: LDL-cholesterol
LPS: lipopolysaccharide
MD2: myeloid differentiation protein 2
MetS: metabolic syndrome
NAFLD: non-alcoholic fatty liver disease
NASH: non-alcoholic steatohepatitis
NLRP: nucleotide-binding oligomerization domain, leucine-rich repeat and pyrin domain-containing protein
Nrf2: nuclear factor erythroid 2-related factor 2
p38: p38 mitogen-activated protein kinase
PEP: phosphoenolpyruvate
PPAR: peroxisome proliferator-activated receptor
ROS: reactive oxygen species
SGLT2: sodium-glucose cotransporter 2
SM934: β-aminoartemether maleate
SOD: superoxide dismutase
SREBP-1c: sterol regulatory element-binding protein 1c
T2DM: type 2 diabetes mellitus
TC: total cholesterol
TCF7L2: transcription factor 7-like 2
TG: triglyceride
Th17: T helper type 17 cell
TLR4: Toll-like receptor 4
Treg: regulatory T cell
WHO: World Health Organization

## References

[1] Alberti KG, Zimmet PZ (1998). Definition, diagnosis and classification of diabetes mellitus and its complications. Part 1: Diagnosis and classification of diabetes mellitus provisional report of a WHO consultation. Diabet Med.

[2] Neeland IJ, Lim S, Tchernof A, Gastaldelli A, Rangaswami J, Ndumele CE, Powell-Wiley TM, Després JP (2024). Metabolic syndrome. Nat Rev Dis Primers.

[3] Son DH, Ha HS, Park HM, Kim HY, Lee YJ (2022). New markers in metabolic syndrome. Adv Clin Chem.

[4] Crecca E, Di Giuseppe G, Camplone C, Vigiano Benedetti V, Melaiu O, Mezza T, Cencioni C, Spallotta F (2025). The multifaceted role of agents counteracting metabolic syndrome: A new hope for gastrointestinal cancer therapy. Pharmacol Ther.

[5] Li H, Ye Z, Zheng G, Su Z (2024). Polysaccharides targeting autophagy to alleviate metabolic syndrome. Int J Biol Macromol.

[6] Saklayen MG (2018). The global epidemic of the metabolic syndrome. Curr Hypertens Rep.

[7] Hou L, Jiang F, Huang B, Zheng W, Jiang Y, Cai G, Liu D, Hu CY, Wang C (2021). Dihydromyricetin ameliorates inflammation—induced insulin resistance via phospholipase C-CaMKK-AMPK signal pathway. Oxid Med Cell Longev.

[8] Barbhuiya PA, Yoshitomi R, Pathak MP (2025). Understanding the link between sterol regulatory element binding protein (SREBPs) and metabolic dysfunction associated steatotic liver disease (MASLD). Curr Obes Rep.

[9] Araújo MC, Soczek SHS, Pontes JP, Marques LAC, Santos GS, Simão G, Bueno LR, Maria-Ferreira D, Muscará MN, Fernandes ES (2022). An overview of the TRP-oxidative stress axis in metabolic syndrome: Insights for novel therapeutic approaches. Cells.

[10] Zhang J, Lv W, Zhang G, Zeng M, Cao W, Su J, Cao K, Liu J (2024). Nuclear factor erythroid 2 related factor 2 and mitochondria form a mutually regulating circuit in the prevention and treatment of metabolic syndrome. Antioxid Redox Signal.

[11] Kalyan M, Tousif AH, Sonali S, Vichitra C, Sunanda T, Praveenraj SS, Ray B, Gorantla VR, Rungratanawanich W, Mahalakshmi AM (2022). Role of endogenous lipopolysaccharides in neurological disorders. Cells.

[12] Mishra S, Jain S, Agadzi B, Yadav H (2025). A cascade of microbiota-leaky gut-inflammation: Is it a key player in metabolic disorders?. Curr Obes Rep.

[13] Saadati S, Mason T, Godini R, Vanky E, Teede H, Mousa A (2025). Metformin use in women with polycystic ovary syndrome (PCOS): Opportunities, benefits, and clinical challenges. Diabetes Obes Metab.

[14] Shi Q, Nong K, Vandvik PO, Guyatt GH, Schnell O, Rydén L, Marx N, Brosius FC, Mustafa RA, Agarwal A (2023). Benefits and harms of drug treatment for type 2 diabetes: Systematic review and network meta-analysis of randomised controlled trials. BMJ.

[15] Hu Y, Guo N, Yang T, Yan J, Wang W, Li X (2022). The potential mechanisms by which artemisinin and its derivatives induce ferroptosis in the treatment of cancer. Oxid Med Cell Longev.

[16] Meng Y, Ma N, Lyu H, Wong YK, Zhang X, Zhu Y, Gao P, Sun P, Song Y, Lin L, Wang J (2021). Recent pharmacological advances in the repurposing of artemisinin drugs. Med Res Rev.

[17] Song C, Huang L, Li D, Zhao X (2025). Artemisinin alleviates alcohol-induced cardiotoxicity by inhibiting ferroptosis via the Nrf2/NQO1 pathway in vivo and in vitro. J Agric Food Chem.

[18] Zhang L, Yu L, Deng D, Liang Q, Du H, Mei M, Deng C, Song J, Guo W (2026). Artemisinin-hydroxychloroquine combination from traditional antimalarial medicine attenuates pulmonary fibrosis via PI3K/Akt/GSK3β-mediated inhibition of epithelial-mesenchymal transition. J Ethnopharmacol.

[19] Posadino AM, Giordo R, Pintus G, Mohammed SA, Orhan IE, Fokou PVT, Sharopov F, Adetunji CO, Gulsunoglu-Konuskan Z, Ydyrys A (2023). Medicinal and mechanistic overview of artemisinin in the treatment of human diseases. Biomed Pharmacother.

[20] Guo X, Asthana P, Zhai L, Cheng KW, Gurung S, Huang J, Wu J, Zhang Y, Mahato AK, Saarma M (2024). Artesunate treats obesity in male mice and non-human primates through GDF15/GFRAL signalling axis. Nat Commun.

[21] Wu T, Feng H, He M, Yue R, Wu S (2022). Efficacy of artemisinin and its derivatives in animal models of type 2 diabetes mellitus: A systematic review and meta-analysis. Pharmacol Res.

[22] Majidiani H, Musavi M, Momtazi-Borojeni AA (2025). New roles of artemisinins in atherosclerosis progression. Phytother Res.

[23] Zhang D, Wu J, Feng H, Tang P, Zhou Y, Zhao C, Liu J, Feng W, Peng C (2025). Gastrodin ameliorates ulcerative colitis via modulating gut microbial tryptophan metabolism and AhR/NLRP3 pathway. Phytomedicine.

[24] Xu J, Huangfu B, Wang T, Ren X, Zhang F, Huang K, He X (2026). Multi-target regulation by artemether in MAFLD through EGFR/HSP90 pathways. J Adv Res.

[25] Meng Q, Zhong M, Zhao J, Xu H, Jiang W, Lin C, Karishma G, Wang F, Xu F, Li Y (2026). Artesunate reduces oxidative stress and inflammation in NAFLD by activating the Nrf2 pathway. Biochem Pharmacol.

[26] Li Y, Yang Y, Li L, Tang K, Hao X, Kai G (2024). Advanced metabolic engineering strategies for increasing artemisinin yield in *Artemisia annua* L. Hortic Res.

[27] Wani KI, Zehra A, Choudhary S, Naeem M, Khan MMA, Khan R, Aftab T (2022). Exogenous strigolactone (GR24) positively regulates growth, photosynthesis, and improves glandular trichome attributes for enhanced artemisinin production in *Artemisia annua*. J Plant Growth Regul.

[28] Dong JX, Dan YM, Tan ZC, Zhao JN, Liu Y (2007). Low temperature molar heat capacities and thermal stability of crystalline artemisinin. Thermochim Acta.

[29] Ho WE, Peh HY, Chan TK, Wong WS (2014). Artemisinins: Pharmacological actions beyond anti-malarial. Pharmacol Ther.

[30] Cao Q, Du H, Fu X, Duan N, Liu C, Li X (2020). Artemisinin attenuated atherosclerosis in high-fat diet-fed ApoE^−^/^−^ mice by promoting macrophage autophagy through the AMPK/mTOR/ULK1 Pathway. J Cardiovasc Pharmacol.

[31] Farmanpour-Kalalagh K, Beyraghdar Kashkooli A, Babaei A, Rezaei A, van der Krol AR (2022). Artemisinins in combating viral infections like SARS-CoV-2, inflammation and cancers and options to meet increased global demand. Front Plant Sci.

[32] Wang S, Wang H, Lin C, Zhang T, Gao J, Wu S, Wang Y, Li H, Min W, Liu C (2021). Structure-activity relationship study of dihydroartemisinin C-10 hemiacetal derivatives as Toll-like receptor 4 antagonists. Bioorg Chem.

[33] Yuan DS, Chen YP, Tan LL, Huang SQ, Li CQ, Wang Q, Zeng QP (2017). Artemisinin: A panacea eligible for unrestrictive use?. Front Pharmacol.

[34] Shen S, Liao Q, Lyu M, Wong YK, Zhang X, Zhou J, Ma N, Wang J (2020). The potential of artemisinins as anti-obesity agents via modulating the immune system. Pharmacol Ther.

[35] Jiang YY, Shui JC, Zhang BX, Chin JW, Yue RS (2020). The potential roles of artemisinin and its derivatives in the treatment of type 2 diabetes mellitus. Front Pharmacol.

[36] Yang J, Huang LJ, Ren TY, Zeng J, Shi YW, Fan JG (2024). Insight into the therapeutic effects of artesunate in relieving metabolic-associated steatohepatitis from transcriptomic and lipidomics analyses. J Dig Dis.

[37] Wang KS, Li J, Wang Z, Mi C, Ma J, Piao LX, Xu GH, Li X, Jin X (2017). Artemisinin inhibits inflammatory response via regulating NF-κB and MAPK signaling pathways. Immunopharmacol Immunotoxicol.

[38] Jiang Y, Du H, Liu X, Fu X, Li X, Cao Q (2020). Artemisinin alleviates atherosclerotic lesion by reducing macrophage inflammation via regulation of AMPK/NF-κB/NLRP3 inflammasomes pathway. J Drug Target.

[39] Madan U, Awasthi A (2025). Antimalarial drug artemotil promotes induction of type 1 regulatory T cells. Inflammation.

[40] Fan Q, Lu H, Chi X, Liu G, Dong Y, Zhang P, Shen X, Fan G (2026). Dihydroartemisinin alleviates ulcerative colitis by modulating gut microbiota and butyrate to restore Treg/Th17 balance and intestinal barrier function. Int Immunopharmacol.

[41] Tong X, Chen L, He SJ, Zuo JP (2022). Artemisinin derivative SM934 in the treatment of autoimmune and inflammatory diseases: Therapeutic effects and molecular mechanisms. Acta Pharmacol Sin.

[42] World Health Organization (2015). Guidelines for the treatment of malaria.

[43] Esu EB, Effa EE, Opie ON, Meremikwu MM (2019). Artemether for severe malaria. Cochrane Database Syst Rev.

[44] Chang Y, Lyu T, Luan X, Yang Y, Cao Y, Qiu Y, Feng H (2025). Artesunate-multiple pharmacological effects beyond treating malaria. Eur J Med Chem.

[45] Fu W, Ma Y, Li L, Liu J, Fu L, Guo Y, Zhang Z, Li J, Jiang H (2020). Artemether regulates metaflammation to improve glycolipid metabolism in db/db mice. Diabetes Metab Syndr Obes.

[46] Zhang Y, Wang Y, Li Y, Huang C, Xiao X, Zhong Z, Tang J, Lu H, Tang Y, Yang J (2022). Dihydroartemisinin and artesunate inhibit aerobic glycolysis via suppressing c-Myc signaling in non-small cell lung cancer. Biochem Pharmacol.

[47] James DE, Stöckli J, Birnbaum MJ (2021). The aetiology and molecular landscape of insulin resistance. Nat Rev Mol Cell Biol.

[48] Chen L, Wang J, Ren Y, Ma Y, Liu J, Jiang H, Liu C (2024). Artesunate improves glucose and lipid metabolism in db/db mice by regulating the metabolic profile and the MAPK/PI3K/Akt signalling pathway. Phytomedicine.

[49] Jain S, Barella LF, Wess J, Reitman ML, Jacobson KA (2021). Adenosine A1 receptor is dispensable for hepatocyte glucose metabolism and insulin sensitivity. Biochem Pharmacol.

[50] Chen W, Tumanov S, Fazakerley DJ, Cantley J, James DE, Dunn LL, Shaik T, Suarna C, Stocker R (2021). Bilirubin deficiency renders mice susceptible to hepatic steatosis in the absence of insulin resistance. Redox Biol.

[51] Guo Y, Fu W, Xin Y, Bai J, Peng H, Fu L, Liu J, Li L, Ma Y, Jiang H (2018). Antidiabetic and antiobesity effects of artemether in db/db mice. Biomed Res Int.

[52] Zhang Y, Lin X, Li J (2023). The controversy about the effects of artemisinins on pancreatic α cell reprogramming and diabetes. Trends Endocrinol Metab.

[53] Efferth T, Oesch F (2021). The immunosuppressive activity of artemisinin-type drugs towards inflammatory and autoimmune diseases. Med Res Rev.

[54] Long Z, Xiang W, Xiao W, Min Y, Qu F, Zhang B, Zeng L (2024). Advances in the study of artemisinin and its derivatives for the treatment of rheumatic skeletal disorders, autoimmune inflammatory diseases, and autoimmune disorders: A comprehensive review. Front Immunol.

[55] Yang C, Zhao X, Zhou W, Li Q, Lazarovici P, Zheng W (2024). Artemisinin conferred cytoprotection to human retinal pigment epithelial cells exposed to amiodarone-induced oxidative insult by activating the CaMKK2/AMPK/Nrf2 pathway. J Transl Med.

[56] Yang C, Xiong W, Dong J, Zhao X, Liang G, Zheng W (2025). Artemisinin protected human bronchial epithelial cells from amiodarone-induced oxidative damage via 5′-AMP-activated protein kinase (AMPK) activation. Redox Rep.

[57] Farhan M, Silva M, Xingan X, Zhou Z, Zheng W (2021). Artemisinin inhibits the migration and invasion in uveal melanoma via inhibition of the PI3K/AKT/mTOR signaling pathway. Oxid Med Cell Longev.

[58] Shaaban HH, Alzaim I, El-Mallah A, Aly RG, El-Yazbi AF, Wahid A (2022). Metformin, pioglitazone, dapagliflozin and their combinations ameliorate manifestations associated with NAFLD in rats via anti-inflammatory, anti-fibrotic, anti-oxidant and anti-apoptotic mechanisms. Life Sci.

[59] Ren Z, Okyere SK, Xie L, Wen J, Wang J, Chen Z, Ni X, Deng J, Hu Y (2022). Oral administration of Bacillus toyonensis strain SAU-20 improves insulin resistance and ameliorates hepatic steatosis in type 2 diabetic mice. Front Immunol.

[60] Xu J, He X, Huang X, Zhang F, Ren X, Asakiya C, Li Y, Huang K (2022). Artemether ameliorates non-alcoholic steatohepatitis by repressing lipogenesis, inflammation, and fibrosis in mice. Front Pharmacol.

[61] Liu P, Wang Y, Tian K, Bai X, Wang Y, Wang Y (2024). Artesunate inhibits macrophage-like phenotype switching of vascular smooth muscle cells and attenuates vascular inflammatory injury in atherosclerosis via NLRP3. Biomed Pharmacother.

[62] Xu J, Cheng X, Wang Q, Zhang F, Ren X, Huang K, Hu Y, Gao R, Yang K, Yin J (2025). Artemether ameliorates non-alcoholic steatohepatitis by restraining cross-talk between lipotoxicity-induced hepatic hepatocytes and macrophages. Phytother Res.

[63] Wang N, Zheng A, Yan Y, He T, Wu X, Xian M, Luo J, Li C, Wei J, Wang Y (2026). Aging induces sarcopenia by disrupting the crosstalk between the skeletal muscle microenvironment and myofibers. J Adv Res.

[64] Zhang Z, Zhang H, Guo H, Zhang X, Wang Y, Wang B, Wang T, Zhao G, Zhang Q, Gao F (2026). Artesunate ameliorates APAP-induced liver injury by promoting NEDD4L-mediated ubiquitination and degradation of TXNIP. Adv Sci (Weinh).

[65] Cui Y, Ling L, Huang Q, Xu H (2023). Artesunate reverses clozapine-induced lipid metabolism disorder in BRL-3A cells by effecting AMPK pathway. Curr Chin Sci.

[66] Yin X, Liu Z, Li C, Wang J (2025). Hinokitiol ameliorates MASH in mice by therapeutic targeting of hepatic Nrf2 and inhibiting hepatocyte ferroptosis. Phytomedicine.

[67] Cen Y, Xiong Y, Qin R, Tao H, Yang Q, Pan X (2023). Anti-malarial artesunate ameliorates atherosclerosis by modulating arterial inflammatory responses via inhibiting the NF-κB-NLRP3 inflammasome pathway. Front Pharmacol.

[68] Jiang W, Cen Y, Song Y, Li P, Qin R, Liu C, Zhao Y, Zheng J, Zhou H (2016). Artesunate attenuated progression of atherosclerosis lesion formation alone or combined with rosuvastatin through inhibition of pro-inflammatory cytokines and pro-inflammatory chemokines. Phytomedicine.

[69] Wang YL, Wang ZJ, Shen HL, Yin M, Tang KX (2013). Effects of artesunate and ursolic acid on hyperlipidemia and its complications in rabbit. Eur J Pharm Sci.

[70] Hinojosa Vera KF, Hemakumar C, Bilachi RS, Ramirez DC, Gomez Mejiba SE (2025). Macrophage phenotypic switch and obesity-associated metabolic risk: Mechanisms and targets. Oxid Med Cell Longev.

[71] Jiménez-Muro M, Soriano-Romaní L, Mora G, Ricciardelli D, Nieto JA (2023). The microbiota-metabolic syndrome axis as a promoter of metabolic osteoarthritis. Life Sci.

[72] Sahariah P, Saikia L, Bharali A, Law D, Sen S, Sivasamugham LA, Dutta PP (2026). Mechanistic insights into natural product-driven modulation of NLRP3-inflammasome signalling in metabolic syndrome. Inflamm Res.

[73] Wijngaarden LH, van der Harst E, Klaassen RA, Dunkelgrun M, Kuijper TM, Klepper M, Ambagtsheer G, IJzermans JNM, de Bruin RWF, Litjens NHR (2021). Effects of morbid obesity and metabolic syndrome on the composition of circulating immune subsets. Front Immunol.

[74] Zhang S, Zhao J, Zhan Y, Li J, Hang J, Tang C, Nong X (2025). Artesunate ameliorates diabetic xerostomia in rats through regulating oral microbiota and metabolic profile in salivary gland based on NF-κB/NLRP3 signaling pathway. Phytomedicine.

[75] Li Q, Yuan Q, Jiang N, Zhang Y, Su Z, Lv L, Sang X, Chen R, Feng Y, Chen Q (2022). Dihydroartemisinin regulates immune cell heterogeneity by triggering a cascade reaction of CDK and MAPK phosphorylation. Signal Transduct Target Ther.

[76] Miao J, Yong Y, Zheng Z, Zhang K, Li W, Liu J, Zhou S, Qin JJ, Sun H, Wang Y (2025). Artesunate inhibits neointimal hyperplasia by promoting IRF4 associated macrophage polarization. Adv Sci (Weinh).

[77] Wang DD, He L, Qi MH, Zhao HY, Yu AX, Huang SW (2025). Mitochondria-targeting artesunate-rhein conjugates: Linker-modulated cell-permeability, heme-affinity and anticancer activity. Eur J Med Chem.

[78] Strik H, Efferth T, Kaina B (2024). Artesunate in glioblastoma therapy: Case reports and review of clinical studies. Phytomedicine.

[79] Li X, Li Q, Jiang N, Zheng K, Zhang Y, Sang X, Feng Y, Chen R, Chen Q (2025). Mechanism of dihydroartemisinin in activating macrophages to enhance host resistance to malaria. Phytomedicine.

[80] Gao S, Chen J, Peng W, Yang Y, Yang Y, Hua L, Guo Y, Wang Y, Zhang X (2023). The preparation and relative bioavailability of an artemisinin self-emulsifying drug delivery system. Drug Deliv.

[81] He W, Du Y, Li C, Wang J, Wang Y, Dogovski C, Hu R, Tao Z, Yao C, Li X (2021). Dimeric artesunate-choline conjugate micelles coated with hyaluronic acid as a stable, safe and potent alternative anti-malarial injection of artesunate. Int J Pharm.

[82] Du X, Liu L, Zhang Y, Zhang B, Yang L, Chu M, Zhou L, Li Y, Zhu J, Dong K (2026). Dihydroartemisinin facilitates intestinal mucosal healing in inflammatory bowel disease by targeting 11βHSD-1 to reprogram macrophage metabolism. Phytomedicine.

[83] Mohaghegh N, Iyer A, Wang E, Balajam NZ, Kang H, Akbari M, Barnhill MS, Khademhosseini A, Pearson RM, Hassani Najafabadi A (2025). Apigenin-loaded nanoparticles for obesity intervention through immunomodulation and adipocyte browning. J Control Release.

[84] Li Z, Samui S, Liu J, Yang Y, Liu X, Chen Q, Li J, Gopinath D, Luo P, Shan D (2026). Gut microbiome and metabolic health: Mechanisms and precision interventions. Gut Microbes.

[85] Das TK, Ganesh BP (2023). Interlink between the gut microbiota and inflammation in the context of oxidative stress in Alzheimer's disease progression. Gut Microbes.

[86] Li Z, Zhou E, Liu C, Wicks H, Yildiz S, Razack F, Ying Z, Kooijman S, Koonen DPY, Heijink M (2023). Dietary butyrate ameliorates metabolic health associated with selective proliferation of gut Lachnospiraceae bacterium 28-4. JCI Insight.

[87] Chen Z, Jia Y, Li H, Fan R, Cao Y, Ni L, Yang L, Yuan Z, Zhu K, Gao Y, Lin Y (2025). Effects of zacopride and multidimensional impacts of cross-kingdom symbiosis: Gut microbiota modulates coronary microvascular dysfunction via the chlorophyll/heme-tryptophan metabolic axis. J Transl Med.

[88] Qi Q, Zhang H, Jin Z, Wang C, Xia M, Chen B, Lv B, Peres Diaz L, Li X, Feng R (2024). Hydrogen sulfide produced by the gut microbiota impairs host metabolism via reducing GLP-1 levels in male mice. Nat Metab.

[89] Liu Y, Yang Y, Lei Y, Yang L, Zhang X, Yuan J, Lei Z (2020). Effects of dihydroartemisinin on the gut microbiome of mice. Mol Med Rep.

[90] Jiang T, Du P, Liu D, Chen H, Ma Y, Hu B, Li J, Jiang H, Li X (2025). Exploring the glucose-lowering and anti-inflammatory immune mechanism of artemether by AMPK/mTOR pathway and microbiome based on multi-omics. Front Pharmacol.

[91] Capatina AL, Czechowski T, Plunkett-Jones C, Tonon T, Kourtzelis I, Lichman BR, Brackenbury WJ, Graham IA, Lagos D (2026). Artemether and Euphorbia factor L9 suppress kynurenine production through distinct effects on tryptophan metabolism. Biochem J.

[92] Pérez-Reytor D, Puebla C, Karahanian E, García K (2021). Use of short-chain fatty acids for the recovery of the intestinal epithelial barrier affected by bacterial toxins. Front Physiol.

[93] Guo Y, Peng Q, Hao L, Ji J, Zhang Z, Xue Y, Liu Y, Gao Y, Li C, Shi X (2022). Dihydroartemisinin promoted FXR expression independent of YAP1 in hepatocellular carcinoma. FASEB J.

[94] Shen H, Ding L, Baig M, Tian J, Wang Y, Huang W (2021). Improving glucose and lipids metabolism: Drug development based on bile acid related targets. Cell Stress.

[95] Zhou H, Huang Y, Chen C, Song M, Hylemon PB (2026). Gut microbiome and bile acid metabolism in liver disease: Mechanisms, clinical implications, and therapeutic opportunities. Pharmacol Rev.

[96] Simbrunner B, Trauner M, Reiberger T (2021). Review article: Therapeutic aspects of bile acid signalling in the gut-liver axis. Aliment Pharmacol Ther.

[97] Guo YQ, Jia CX, Zhu TL, Sun Z, Xi YT, Liu X, Li CC, Li P, Li CZ, Niu QQ (2026). Mechanism and interventions of the bile acid-gut-liver axis imbalance in the progression of non-alcoholic fatty liver disease. Biochem Pharmacol.

[98] Chen J, He X, Bai Y, Liu J, Wong YK, Xie L, Zhang Q, Luo P, Gao P, Gu L (2023). Single-cell transcriptome analysis reveals the regulatory effects of artesunate on splenic immune cells in polymicrobial sepsis. J Pharm Anal.

[99] Xun Z, Yao X, Lin C, Yang X, Zhang Y, Yang X, He Y, Jiang R, Lan Y, Ye Y (2025). A novel therapy targeting the gut-liver axis for chronic hepatitis B: Ursodeoxycholic acid plus *Bifidobacterium*. JHEP Rep.

[100] Ahmad K, Shaikh S, Lim JH, Ahmad SS, Chun HJ, Lee EJ, Choi I (2023). Therapeutic application of natural compounds for skeletal muscle-associated metabolic disorders: A review on diabetes perspective. Biomed Pharmacother.

[101] Liu F, Chen J, Wu T, Xu H, Zhuang S, Ma X, Yue H, Dai Y (2026). Amelioration of diabetic myopathy by APMCG-1: A mountain-cultivated ginseng-derived glycopeptide evaluated in zebrafish and mouse models. J Ethnopharmacol.

[102] Pan J, Zhang Y, Du Z, Huang Y, Wang Y, Su M, Cai Y, Xu W, Chen L, Huang C (2026). Artemisinin derivative SM934 targets α-enolase to inhibit PEP-mediated TAK1 stabilization and inflammation. Sci China Life Sci.

[103] Yang FM, Fan D, Yang XQ, Zhu FH, Shao MJ, Li Q, Liu YT, Lin ZM, Cao SQ, Tang W (2021). The artemisinin analog SM934 alleviates dry eye disease in rodent models by regulating TLR4/NF-κB/NLRP3 signaling. Acta Pharmacol Sin.

[104] Mu L, Shen Y, Wang G, Liu C, Yang H, Li W, Mol BW, Li X, Dong X, Jiang J (2026). Dihydroartemisinin for the treatment of polycystic ovary syndrome: Study protocol for a multi-centre placebo-controlled randomised clinical trial. BMJ Open.

[105] Hu Y, Peng X, Du G, Zhai Y, Xiong X, Luo X (2023). Dihydroartemisinin ameliorates the liver steatosis in metabolic associated fatty liver disease mice by attenuating the inflammation and oxidative stress and promoting autophagy. Acta Cir Bras.

[106] Efferth T, Kaina B (2010). Toxicity of the antimalarial artemisinin and its derivatives. Crit Rev Toxicol.

[107] Gordi T, Lepist EI (2004). Artemisinin derivatives: Toxic for laboratory animals, safe for humans?. Toxicol Lett.

[108] Jiang B, Wei JX, Liu XC, He L, Hou T, Niu B (2026). Safety profile of artemether: Analysis based on 15-year data retrieved from the FDA Adverse Event Reporting System (FAERS). Asian Pac J Trop Med.

[109] Burk O, Arnold KA, Nussler AK, Schaeffeler E, Efimova E, Avery BA, Avery MA, Fromm MF, Eichelbaum M (2005). Antimalarial artemisinin drugs induce cytochrome P450 and MDR1 expression by activation of xenosensors pregnane X receptor and constitutive androstane receptor. Mol Pharmacol.

[110] Hernandez Maldonado J, Grundmann O (2022). Drug-drug interactions of artemisinin-based combination therapies in malaria treatment: A narrative review of the literature. J Clin Pharmacol.

[111] van der Pluijm RW, Amaratunga C, Dhorda M, Dondorp AM (2021). Triple artemisinin-based combination therapies for malaria-a new paradigm?. Trends Parasitol.

[112] Noubiap JJ, Nansseu JR, Nyaga UF, Ndoadoumgue AL, Ngouo AT, Tounouga DN, Tianyi FL, Foka AJ, Lontchi-Yimagou E, Nkeck JR, Bigna JJ (2025). Worldwide trends in metabolic syndrome from 2000 to 2023: A systematic review and modelling analysis. Nat Commun.

[113] Miyashita Y, Hitsumoto T, Fukuda H, Kim J, Ito S, Kimoto N, Asakura K, Yata Y, Yabumoto M, Washio T, Kitakaze M (2023). Metabolic syndrome is linked to the incidence of pancreatic cancer. EClinicalMedicine.

[114] Zhou L, Gao H, Zhang J, Xu Q, Wang Q, Wang L, Tan Y, Luo Z, Zhou J, Shuai H (2025). Metabolic syndrome and cancer risk: A two-sample Mendelian randomization study of European ancestry. Int J Surg.

[115] Rosenthal PJ, Asua V, Conrad MD (2024). Emergence, transmission dynamics and mechanisms of artemisinin partial resistance in malaria parasites in Africa. Nat Rev Microbiol.

[116] Watson DJ, Laing L, Gibhard L, Wong HN, Haynes RK, Wiesner L (2021). Toward new transmission-blocking combination therapies: Pharmacokinetics of 10-amino-artemisinins and 11-aza-artemisinin and comparison with dihydroartemisinin and artemether. Antimicrob Agents Chemother.

[117] Gibhard L, Coertzen D, Reader J, van der Watt ME, Birkholtz LM, Wong HN, Batty KT, Haynes RK, Wiesner L (2021). The artemiside-artemisox-artemisone-M1 tetrad: Efficacies against blood stage P. falciparum parasites, DMPK properties, and the case for artemiside. Pharmaceutics.

